# RNA-Seq and Gene Network Analysis Uncover Activation of an ABA-Dependent Signalosome During the Cork Oak Root Response to Drought

**DOI:** 10.3389/fpls.2015.01195

**Published:** 2016-01-11

**Authors:** Alexandre P. Magalhães, Nuno Verde, Francisca Reis, Inês Martins, Daniela Costa, Teresa Lino-Neto, Pedro H. Castro, Rui M. Tavares, Herlânder Azevedo

**Affiliations:** ^1^BioSystems and Integrative Sciences Institute, Plant Functional Biology Center, University of MinhoBraga, Portugal; ^2^CIBIO, InBIO – Research Network in Biodiversity and Evolutionary Biology, Universidade do PortoVairão, Portugal

**Keywords:** ABA, ABF, drought, *Quercus suber*, RNA-Seq, root transcriptomics

## Abstract

*Quercus suber* (cork oak) is a West Mediterranean species of key economic interest, being extensively explored for its ability to generate cork. Like other Mediterranean plants, *Q. suber* is significantly threatened by climatic changes, imposing the need to quickly understand its physiological and molecular adaptability to drought stress imposition. In the present report, we uncovered the differential transcriptome of *Q. suber* roots exposed to long-term drought, using an RNA-Seq approach. 454-sequencing reads were used to *de novo* assemble a reference transcriptome, and mapping of reads allowed the identification of 546 differentially expressed unigenes. These were enriched in both effector genes (e.g., LEA, chaperones, transporters) as well as regulatory genes, including transcription factors (TFs) belonging to various different classes, and genes associated with protein turnover. To further extend functional characterization, we identified the orthologs of differentially expressed unigenes in the model species *Arabidopsis thaliana*, which then allowed us to perform *in silico* functional inference, including gene network analysis for protein function, protein subcellular localization and gene co-expression, and *in silico* enrichment analysis for TFs and *cis*-elements. Results indicated the existence of extensive transcriptional regulatory events, including activation of ABA-responsive genes and *ABF*-dependent signaling. We were then able to establish that a core ABA-signaling pathway involving *PP2C*-*SnRK2*-*ABF* components was induced in stressed *Q. suber* roots, identifying a key mechanism in this species’ response to drought.

## Introduction

Cork oak (*Quercus suber*) is an evergreen species of the Fagaceae family, and one of the most significant forest species in the Mediterranean region, due to both its ecological dominance and economic value (Nixon, 1993; Belahbib et al., 2001). *Q. suber* is a slow growing, extremely long-lived tree, reaching a height of up to 20 m, with massive branches forming a round crown (Faria et al., 1996). The modern distribution of cork oak is rather discontinuous, ranging from the Atlantic coasts of North Africa and Iberian Peninsula to the south-eastern regions of Italy, Sicily and Sardinia, as well as the coastal belts of Algeria and Tunisia, France and Spain (Lumaret et al., 2005; Magri et al., 2007). As a species that is endemic to the Western Mediterranean region, *Q. suber* is mostly present in semi-natural stands known as *Montados*, which are open woods with a delicate and particular ecosystem, created and maintained by man (Sánchez-González et al., 2008). *Montados* and cork oak management represent an important economical resource, as they are associated not only with the harvesting of acorns, but also with the use of bark as the source of cork (Lopes et al., 2001). Presently, *Q. suber* forests cover 2.2 million hectares worldwide, providing 340,000 tons/year of cork. Yet, *Montados* are currently threatened and in decline due to multiple factors, the main of which is the occurrence of severe drought periods over several consecutive years (Toumi and Lumaret, 1998). As opposed to other oaks which have a greater ecological amplitude, this species is a sclerophyllous tree that is adapted to a 4-month-long hot-dry summer period, with at least 450 mm mean annual rainfall (Faria et al., 1996; Toumi and Lumaret, 1998). Mediterranean-climate regions are characterized by a cycle of temperatures out of phase with the rainfall, producing mild to cool rainy winters and dry summers. However, the proneness for hydrological variability of Mediterranean climate regions make these areas particularly sensitive to global climatic changes, and an increase in drought period frequency is expected in forthcoming years. These events are likely to have a significant impact on *Q. suber* distribution, sustainability and commercial exploitation.

Mediterranean vegetation dealing with the peculiar soil moisture dynamics of this region, developed a number of physiological mechanisms to tolerate drought stress and growth under adverse climatic conditions. Mechanisms include early responses involving stomatal closure, to prevent or delay tissue dehydration, and antioxidant biosynthesis as a photoprotection mechanism (Flexas et al., 2014). Long term acclimation responses include decreased growth, to reduce water and nutrient demands (Nuche et al., 2014), changes in allocation of resources from support tissues to assimilating organs (Nuche et al., 2014), and development of strategies to prevent xylem cavitation, like refilling mechanisms, regulation of hydraulic conductance, and xylem margin reinforcement and repair (Nardini et al., 2014). At the molecular level, plants have developed numerous advanced response mechanisms to cope with the challenges facing a sessile organism, including microRNA regulation (Lu and Huang, 2008; Khraiwesh et al., 2012), chromatin remodeling (Golldack et al., 2011; Luo et al., 2012) and examples of post-translational modification of proteins (Castro et al., 2012). However, the best-characterized mechanisms involve gene expression regulation, engaging signal transduction pathways to trigger changes in metabolic processes, management of resources, and organ morphology (Wang et al., 2003; Danquah et al., 2014). Gene regulatory pathways include environmental sensing mechanisms, membrane-localized elements, signaling transduction components such as MAP kinase cascades, hormone-dependent signaling modules, and induction of several classes of transcription factors (TFs), such as ABF, AP2/ERF, NAC, MYB, DREB/CBF and HSF TFs (Wang et al., 2003, 2004; Yoshida et al., 2010; Lata and Prasad, 2011; Atkinson and Urwin, 2012; Chen et al., 2012; Mizoi et al., 2012; Nakashima et al., 2012, 2014; Osakabe et al., 2013; Danquah et al., 2014). In drought stress resistance, four major signaling pathways have been described, involving both ABA-dependent and ABA-independent signal transduction, and ABF, MYC, DREB and NAC TFs. The foremost, best characterized signaling pathway, also designated the core ABA transduction pathway, involves a signalosome composed of several components, namely PYR/PYL/RCAR (ABA receptor), PP2C (type 2C protein phosphatases) and SnRK2s (SNF1-related protein kinase 2), and is traditionally associated with the regulation of ABF TFs (Kuromori et al., 2014). These regulatory events manage the expression of functional downstream response genes, involved in growth regulation, water management, ROS scavenging and induction of secondary metabolism (Wang et al., 2003).

Next generation sequencing (NGS) technology is revolutionizing our capacity to perform research in various areas of the biological field. The ability to generate massive sequencing libraries with a lower cost and high degree of performance has profoundly affected present research strategies, facilitating studies in non-model systems and easily moving species toward an *Omics* age. In plants, the number of genomes being sequenced has been consistently increasing, and accompanying this trend, RNA-sequencing (RNA-Seq) has also provided a massive increase in the number of information generated by NGS. This method has been replacing other transcriptomics methodologies as the main research tool for RNA expression profiling, providing high resolution transcriptomes capable of quantifying whole-genome expression more accurately and at higher detection limits (Martin et al., 2013). Possibly the most important recent development in *Q. suber* research was the Portuguese nation-wide initiative, the *Cork oak ESTs Consortium* (COEC), which performed 454 *de novo* sequencing of the *Q. suber* transcriptome. It used cDNA samples derived from 21 normalized libraries, representing multiple tissues, organs, developmental stages and physiological/environmental conditions (Pereira-Leal et al., 2014). Within this framework, a number of parallel projects were carried out with the objective of analyzing differential expression of the *Q. suber* transcriptome on non-normalized libraries (Rocheta et al., 2014; Sebastiana et al., 2014; Teixeira et al., 2014). Here, we report one such study, which addressed the transcriptional reprogramming of *Q. suber* root tissues in response to long-term water deficit. This RNA-Seq approach allowed identification of over 500 differentially expressed unigenes, including a significant number of different TFs and other signal transduction components. In order to functionally characterize the differential transcriptome, an *in silico*, homology-based gene network analysis was performed, ultimately uncovering the activation of a complete canonical ABA/ABF-dependent signaling pathway in *Q. suber* roots responding to long-term drought stress.

## Materials and Methods

### Plant Material and Physiological Assessments

*Quercus suber* acorns were supplied by Instituto Superior de Agronomia (ISA, Lisbon). Acorns were stratified at 4°C for 2 weeks and subsequently sowed in a soil mixture consisting of a 2:1:1 volume mix of turf, vermiculite and silicate sand. A total of 123 vases were sowed and grown in a 16 h light/8 h dark photoperiod regime (100 μmol Photon m^-2^ s^-1^). After 80 days, drought treatments were initiated. Treatments used 90 plants presenting the best fitness, divided into 18 groups, with five plants each. Plants from each group were subjected to five different drought treatments, corresponding to 100, 90, 50, 25, and 10% of the field capacity of the soil mixture, respectively identified as D100, D90, D50, D25, and D10. Watering was performed three times a week. In each group, plants were watered with 100, 90, 50, 25, or 10% of the pot weight loss measured in D100 samples. Quantum yield of photosystem II (Fv/Fm) was assessed by pulse amplitude modulated (PAM) fluorometry, after adapting plants to the dark for 15 min, using a portable Pulse Amplitude Modulated fluorometer (Junior-PAM, Gademann Instruments GmbH, Germany). Blue light was used as the light source. For each condition, three healthy leaves were selected in three independent plants, and Fv/Fm was evaluated throughout the drought assay. Chlorophyll and carotenoid pigments were quantified spectrophotometrically as previously reported (Hipkins and Baker, 1986). Quantification of L-proline levels in roots was performed using the ninhydrin method, as previously reported (Bates et al., 1973). Commercially available L-Proline was used for the standard curve.

### 454 Sequencing

Total RNA was isolated from secondary roots. These were snap-frozen and grinded in liquid nitrogen. Total RNA was extracted following the Hot Borate method (Wan and Wilkins, 1994) and further purified using the RNeasy Plant Mini Kit (Qiagen). Sequencing was carried out in a Genome Sequencer GS FLX Titanium (Roche-454 Life Sciences, Brandford, CT, USA) according to the manufacturer’s instructions, at Biocant (Cantanhede, Portugal). Each library (D100/D90, D50 and D25/10) occupied one half of a picotiter plate. Raw reads were subsequently subjected to trimming, removal of adapters/primers and low quality reads using SeqTrim (minimum read length = 50 bp; number of error allowed in MID = 2; average limit filter quality = 13; size window filter quality = 7; max undetermined nucleotides = 2) (Falgueras et al., 2010). Sequences were deposited in the National Center for Biotechnology Information (NCBI), under the Short Read Archive (SRA) accession number SRP055382.

### Sequence Assembly and Differential Expression Analysis

*De novo* assembly was carried out using Newbler (GS De Novo Assembler V2.9) with default parameters and the resulting assembly file was submitted to the CD-HIT-EST web server (95% identity; Huang et al., 2010) to eliminate redundancies originated from the *de novo* assembly. Sequences with less than 100 bp were removed and the remaining isotigs and contigs were named *Unigenes*. Unigenes were given an accession number using an in-house Perl script. Fasta sequences are available in **Supplementary File S1**. Unigenes were subjected to homology search using BLASTx (BLASTX 2.2.28+) against the non-redundant protein database (nr) with an *E*-value threshold of 1e-6. GI numbers were extracted and submitted to Uniprot’s Retrived/ID mapping tool^1^ to generate GO enrichment. Coverage for the assembled transcriptome was assessed with BLASTx (BLASTX 2.2.28+) analysis, against the *Castanea molissima* predicted transcriptome and *Quercus rubra* Unigene V2 transcriptome from Fagaceae Genomics Web^2^, and *A. thaliana* predicted transcriptome^3^, with an *E*-value threshold of 1e-6.

To determine the set of differentially expressed genes (DEGs) between the three experimental conditions, high quality (HQ) reads from each sequencing effort were mapped against the assembled transcriptome using the Burrows–Wheeler Aligner (BWA^4^). The algorithm chosen was BWA-MEM as it features long-read support and split alignment that is suitable for 454 reads, and is faster and more accurate when compared to BWA-SW. Parameters were set to promote single alignments for each read (-c command with parameter of “1”). Summarization of results was performed using Tablet (Milne et al., 2012). Venn diagrams were generated using Venny v2.0^5^. Identification of DEGs was performed in DESeq (Anders and Huber, 2010). Using DESeq, we modeled count data using a negative binomial distribution, paring all conditions (D100/D90 vs. D50; D25/D10 vs. D50; D100/D90 vs. D25/D10). We retained unigenes that displayed differential expression (significance value < 0.05) in at least one comparison. In the absence of replicates, we used DESeq’s blind method for dispersion estimates: FDR correction was disabled and Padj reported as “1”. CDBFasta^6^ was used to retrieve specific Fasta sequences from the assembled unigenes file. For Hierarchical clustering (HCL; Eisen et al., 1999), normalized read counts for each DEG were retrieved from DESeq; HCL was performed by Euclidean distance and average linkage clustering on Multiple Experiment Viewer (MeV v4.0^7^). Gene clusters were identified using the MeV feature Self Organizing Tree Algorithm (SOTA; Herrero et al., 2001), using Euclidean distance. Expression plots were rendered considering SOTA clustering, and DEGs were separated into up-regulated and down-regulated genes based on the Expression plot profile.

### Protein Prediction, Functional Annotation and Ortholog Assessment

BLASTx results for the differentially expressed unigenes (**Supplementary File S2**) were extracted from the previously established BLASTx results of the whole transcriptome (see Sequence Assembly and Differential Expression Analysis). The ORF Predictor^8^ tool was used to identify open reading frames (ORFs). For this purpose, BLASTx results and nucleotide sequences were uploaded to the ORF Predictor server, so frame shift errors and incomplete sequences could be accounted for. Corrected CDS and protein sequences were retrieved for later analysis. Nucleotide sequences were also analyzed using the Blast2GO pipeline (level 3; Conesa et al., 2005), using standard settings and the local BLASTx previously generated. Gene subsets were created within the Blast2GO software, comprising the genes assigned to each cluster defined earlier by SOTA analysis. GO enrichment analysis for *Biological process* was then carried out on each gene cluster individually, using an *E*-value threshold of 1e-5. To establish *A. thaliana* orthologs, DEG protein sequences were subjected to BLASTp analysis (*E*-value of 1e-6) against the reference *A. thaliana* TAIR10 annotation^9^. Only the gene model was considered for later analysis.

### Functional Data Mining in *Arabidopsis* and Validation by qPCR

Transcription factors were identified from *Arabidopsis* DEG orthologs using the AGRIS *Arabidopsis* TF database (AtTFDB^10^; Davuluri et al., 2003), MapMan (transcripton pathway bin; Thimm et al., 2004), and manual curation. *Cis*-element enrichment analysis was carried using AtCOECis (Vandepoele et al., 2009) and Athena (O’Connor et al., 2005), with default parameters. Gene networks were predicted in Cytoscape, using the Genemania App (Warde-Farley et al., 2010; Saito et al., 2012) (Supplementary Methods S1). Phylogenetic reconstruction was carried out by maximum likelihood, after automated retrieval of *Arabidopsis* TF gene family members (Supplementary Methods S2). For quantitative Real-Time PCR (qPCR) analysis, a second independent drought induction assay was performed, and total RNA was isolated from secondary roots by a CTAB-based method adapted from Azevedo et al. (2003). Validation of differential expression by qPCR was performed according to standard procedures (Supplementary Methods S3), using primer sequences described in Supplementary Table S1.

## Results and Discussion

### Physiological Response to Long-Term Drought

**FIGURE 1:**
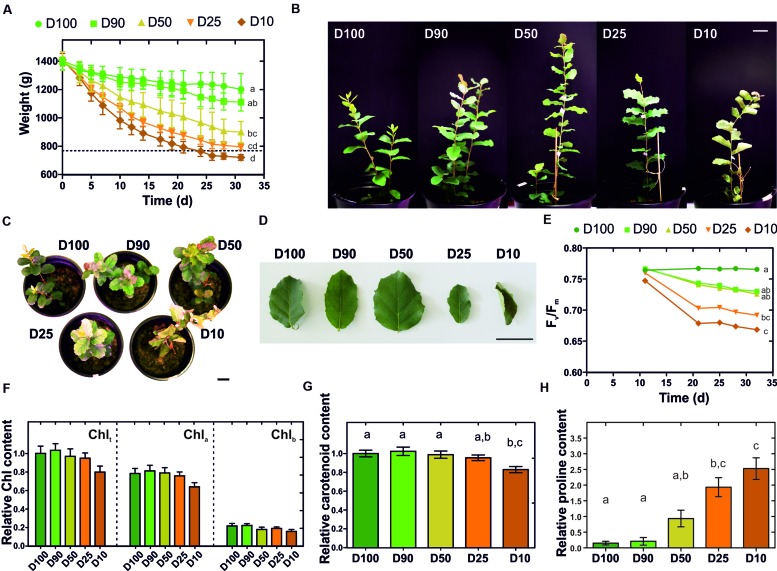
**Characterization of morphological and physiological traits of *Q. suber* plantlets subjected to long-term drought stress. (A)** Eighty-day-old plantlets were subjected to five different watering regimens (D100, D90, D50, D25, D10) for one month; pot weight was determined before each watering period, (*n* = 6). **(B–D)** Plant **(B,C)** and leaf **(D)** morphology at the end of the drought stress period. **(E)** Effect of each watering regimen in the quantum yield of photosystem II (Fv/Fm), (*n* = 9). **(F)** Total chlorophyll (Chl_t_), chlorophyll a (Chl_a_) and chlorophyll b (Chl_b_) contents at the end of the drought stress period, (*n* = 15). **(G)** Carotenoid content at the end of the drought stress period, (*n* = 15). **(H)** Quantification of L-proline in *Q. suber* roots at the end of the drought stress period, (*n* = 15). Letters represent statistically similar results (one-way ANOVA with *post hoc* Tukey test to compare all columns; *p* > 0.05). Scale bars represent 3 cm.

The cork oak root-level response to drought was investigated at the transcriptional level by an RNA-Seq approach. *Q. suber* plantlets were subjected to prolonged drought stress and characterized in their morphological and physiological responses. More specifically, 2-month-old plantlets were subjected for 1 month to five different watering regimes (herein designated D100, D90, D50, D25, and D10), that restored 100, 90, 50, 25, or 10% of the water lost between watering periods. This imposed a progressive loss in water availability, resulting in moderate to extreme drought stress over an extended period of time (**Figure 1A**). The consequences of drought stress imposition were visible in leaf morphology, with leaves displaying increasing stress-related symptoms such as leaf area drop, leaf rolling, edge and tip necrosis, and changes in coloration observable as reddening and yellowing (**Figures 1B–D**). To support morphological data, alterations to plant fitness throughout the treatment period were monitored using PAM fluorometry. This technique has been successfully used to study alterations in photosynthetic electron transport *in vivo* (Papageorgiou, 2007). A progressive loss in efficiency of PSII, determined by the F_v_/F_m_ parameter (maximum quantum efficiency of PSII photochemistry) was detected for all major drought treatments, especially for D25 and D10 (**Figure 1E**). This may be related to inhibition of the photosynthetic electron transport activity or down-regulation of metabolic activity, resulting in a reduction in CO_2_ assimilation which is associated with drought-stress response in plants (Li et al., 2006). Also, long-term drought stress can result in physical destabilization and reorganization of the PSII (Giardi et al., 1996), therefore the decrease in F_v_/F_m_ could be related to such events. Our results are also consistent with other studies in oak species undergoing water deprivation, in which a decrease in F_v_/F_m_ was observed (Epron et al., 1993; Méthy et al., 1996). Additionally, we estimated chlorophyll and carotenoid contents in leaves at the end of the experiment, observing a reduction in both pigments that was proportional to the severity of the drought treatment (**Figures 1F,G**). Overall results are consistent with previous reports that associate molecular and physiological responses to water deficit with chlorophyll bleaching, decline in photosynthesis, accumulation of carotenoid-like molecules, remobilization of nutrients, dismantling of cellular organelles, and programmed cell death events (Shao et al., 2008). Finally, to confirm drought stress imposition we quantified the root’s proline content. Proline is a standard indicator of low water potential (Ψ_w_) caused by environmental stresses, acting as a compatible solute that accumulates to high concentrations following decreases in water availability (Lambers et al., 2008). A significant and dose dependent increase in the proline content was detected in drought-stressed *Q. suber* roots (**Figure 1H**), thus confirming the imposition of drought stress. In sum, we clearly established that in our physiological model, different watering regimes matched different intensities of long-term drought stress imposition.

### Assembly of the *Q. suber* Root Transcriptome

**FIGURE 2:**
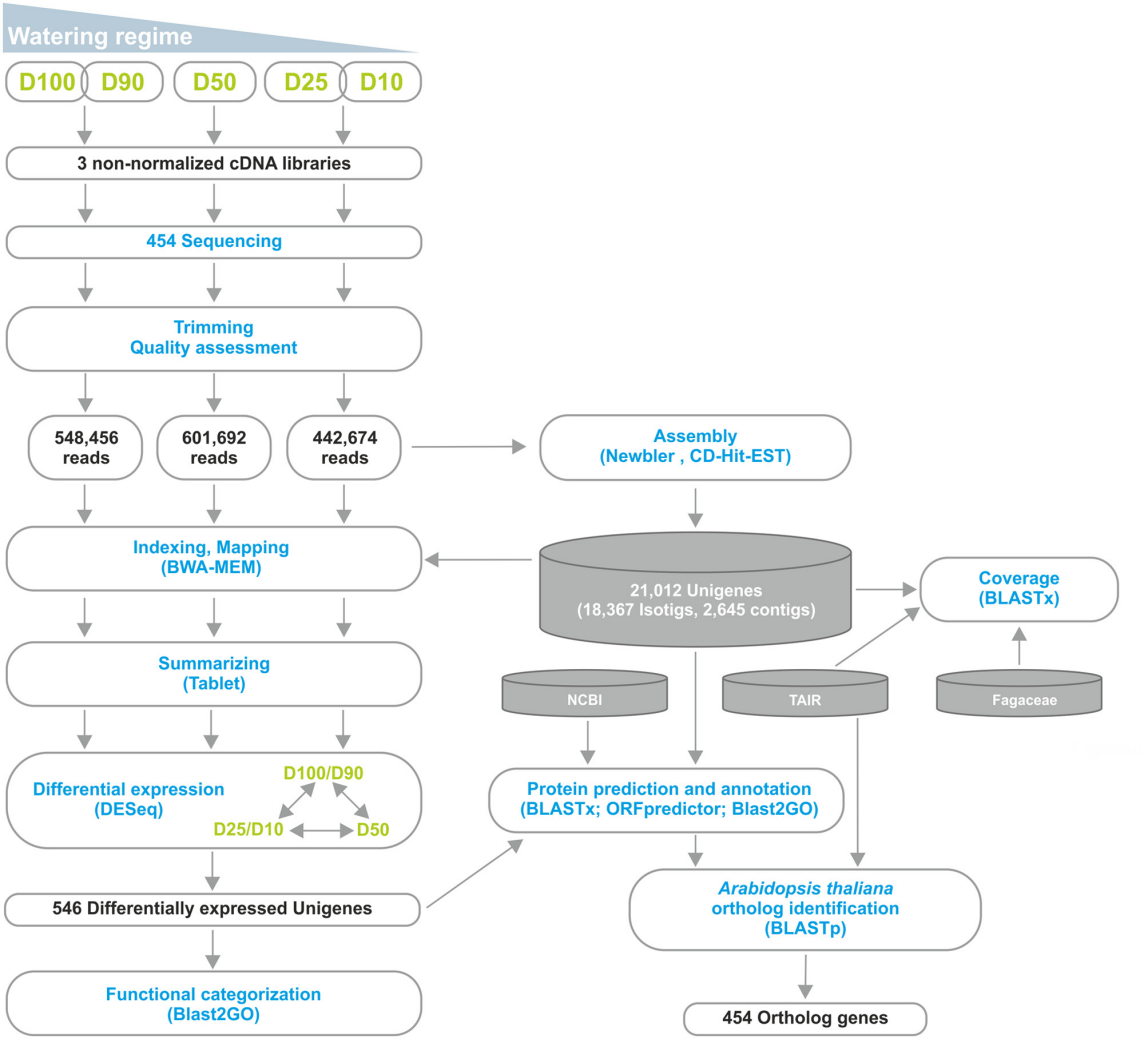
**Outline of the RNA-Seq strategy employed in the identification of the *Q. suber* differential root transcriptome in response to long-term drought.** Functional characterization was extended by identification of *Arabidopsis thaliana* orthologs for the differentially expressed genes.

We were involved in a Portuguese nation-wide initiative, the COEC, that performed *de novo* sequencing of the *Q. suber* transcriptome by 454 NGS (^11^Pereira-Leal et al., 2014). Within this initiative, we performed a parallel RNA-Seq analysis of the *Q. suber* root response to long term drought. RNA was extracted from roots of plantlets subjected to different watering conditions (D100+D90, D50 and D25+D10), and used to generate non-normalized libraries that were then sequenced by 454 GS FLX Titanium technology, using half-plate runs for each experimental condition (**Figure 2**). Results from the sequencing effort are presented in **Table 1**. Raw read number (1.8 million) and length (∼400 bp) followed expected standards for this technology. Raw reads were subjected to a pre-processing/trimming step to remove short or low quality sequences and adaptor/primer sequences. HQ reads amounted to 88.1% of original reads, and totaled 470.8 Mbp (**Table 1**). HQ reads displayed no significant differences in number or length frequency, between different drought stress regimes (**Table 1**; Supplementary Figure S1A), corroborating the quality of the cDNA library and sequencing processes. Subsequently, we assembled a reference transcriptome using HQ reads, resulting in a total of 22,455 raw sequences; these were uploaded to the CD-HIT-EST web server (Huang et al., 2010) for clustering and redundancy elimination, resulting in 21,012 unigenes of which 18,367 were isotigs and 2,645 were contigs (**Figure 2**, **Table 1**). Frequency analysis demonstrated that the assembled unigenes were as long as 2,600 bp, averaged at 758 bp and peaked in the 400–500 bp range (Supplementary Figure S1B). Of the total amount of reads that could be aligned, 70% had a HQ alignment score. Gene Ontology (GO) category enrichment was calculated for the whole transcriptome (Supplementary Figure S1C), demonstrating a foreseeable abundance in metabolic and cellular processes, as well as catalytic and binding protein functions.

**Table 1:** Statistics of the sequencing and assembly steps.

	D100/D90	D50	D25/D10	Total
**Raw reads**				
Number	686,731	620,463	501,296	1,808,490
Total number of bases (Mbp)	270.9	250.8	198.5	720.3
Average length (bp)	394.6	404.3	396.0	398.3
**Processed reads**				
Number	548,456	601,692	442,674	1,592,822
Total number of bases (Mbp)	164.0	172.4	134.4	470.8
Average length (bp)	299.0	286.5	303.7	296.4
**Assembly**				
Number of contigs	–	–	–	2,645
Number of isotigs	–	–	–	18,367
Number of isogroups	–	–	–	19,579
Number of unigenes	–	–	–	21,012

**FIGURE 3:**
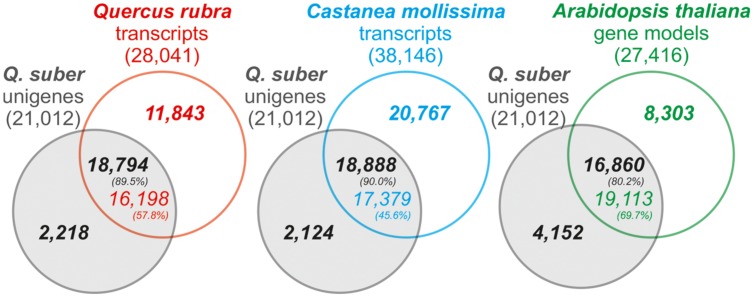
**Venn diagram representation of the number of homology hits (*E*-value < 1e-6) between *Q. suber* unigenes and *Arabidopsis thaliana* TAIR 10 gene models, *Quercus rubra* putative transcripts and the *Castanea mollissima* predicted transcriptome.** Percentages indicate the proportion of matching genes between reference transcriptomes and *Q. suber* unigene models.

For quality validation, the assembled transcriptome’s coverage was established against the *Quercus rubra* Unigene V2 transcriptome, *Castanea molissima* predicted transcriptome, and *A. thaliana* transcriptome, as well as the *Q. suber* total transcriptome, previously established by COEC (Pereira-Leal et al., 2014; herein *total transcriptome*). Results demonstrated that the present *Q. suber* transcriptome had a very high coverage, with 80–90% unigenes matching 45–70% of transcriptomes from other species (**Figure 3**). Furthermore, 98.5% of unigenes were present in the previous total transcriptome (Supplementary Figure S2). In support of the present strategy to perform a *de novo* drought-associated transcriptome assembly, we observed that our N50 for transcript length (758 bp) was over 4 times higher than that of the total transcriptome (N50 = 179 bp), and ∼20 thousand unigenes matched over 80 thousand transcripts of the total transcriptome, indicating that present unigenes are proportionally representing contigs that failed to assemble in the total transcriptome. Moreover, the present N50 value is similar to equivalent recent *Q. suber* transcriptomes that also adopted a *de novo* assembly strategy (N50 = 689 bp, Sebastiana et al., 2014; N50 = 914 bp, Rocheta et al., 2014; N50 = 770 bp, Teixeira et al., 2014). Our results further suggest that Newbler-based assembly (present report; Rocheta et al., 2014; Teixeira et al., 2014) is more suited for 454 libraries than MIRA-based assembly (Pereira-Leal et al., 2014; Sebastiana et al., 2014), since the latter studies presented poorer metrics (lower N50, significantly higher unigene number). This evidence supports the previous claim that Newbler outperforms other assemblers with regards to 454 pyrosequencing data (Kumar and Blaxter, 2010).

### Differential Expression Analysis of the Drought Root Transcriptome

**FIGURE 4:**
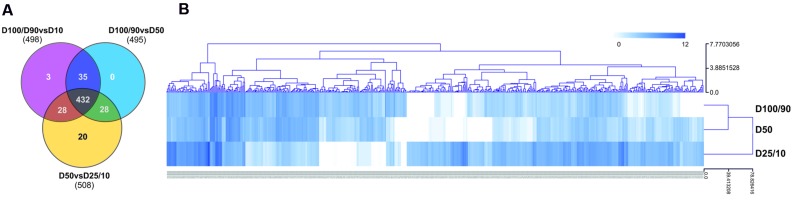
**Characterization of differential gene expression in D100/90 vs. D50, D100/90 vs. D25/10 and D50 vs. D25/10 transcriptional comparisons. (A)** Venn diagram representing unigenes in each experimental comparison. **(B)** Hierarchical clustering analysis of DEG gene expression values.

For differential expression analysis, HQ reads from each sample were mapped to the reference transcriptome (**Figure 2**). Distribution analysis for the number of reads mapped to each unigene demonstrated that approximately 98% of unigenes displayed a fairly low number of reads (<100; Supplementary Figure S3A). Consistently, the different watering regimes showed similar distribution profiles. To establish differential expression, unigenes were then subjected to read count differential analysis by Negative Binomial Distribution (Anders and Huber, 2010). Briefly, all three experimental situations were paired for differential expression and genes that displayed *p*-value <0.05 in at least one comparison were selected, resulting in a total of 546 DEGs (**Figures 2** and **4**). Interestingly, comparative analysis showed that most DEGs (79%) were expressed differentially throughout the three transcriptional pools (**Figure 4A**), suggesting the existence of dramatic/extensive transcriptional rearrangement between control, moderately- and severely stressed plants. HCL analysis was subsequently performed on DEGs (**Figure 4B**). Based on expression patterns, we differentiated nine gene clusters containing 353 up-regulated and 193 down-regulated unigenes (**Figure 4B**; Supplementary Figures S3B,C). Clusters of up-regulated genes were enriched in *response to stress* GO categories, whereas clusters of down-regulated genes were enriched in *transport and energy pathway*-related genes (Supplementary Figure S3C). In response to long term drought, up-regulated genes surpassed down-regulated genes by over twofold. Results support that the present transcriptomics strategy was efficient in resolving transcriptional changes. Data specifically suggests that the switch from moderate to severe drought (*D50 vs. D25/D10*) was transcriptionally more dramatic, considering that it evidenced (1) the highest number of unique DEGs (**Figure 4A**), (2) the most scattered pattern in MA plot projections (Supplementary Figure S3D), (3) the most significant differences in fold change values (**Figure 4B**; Supplementary Figure S3B).

### Annotation of Differentially Expressed Genes

**FIGURE 5:**
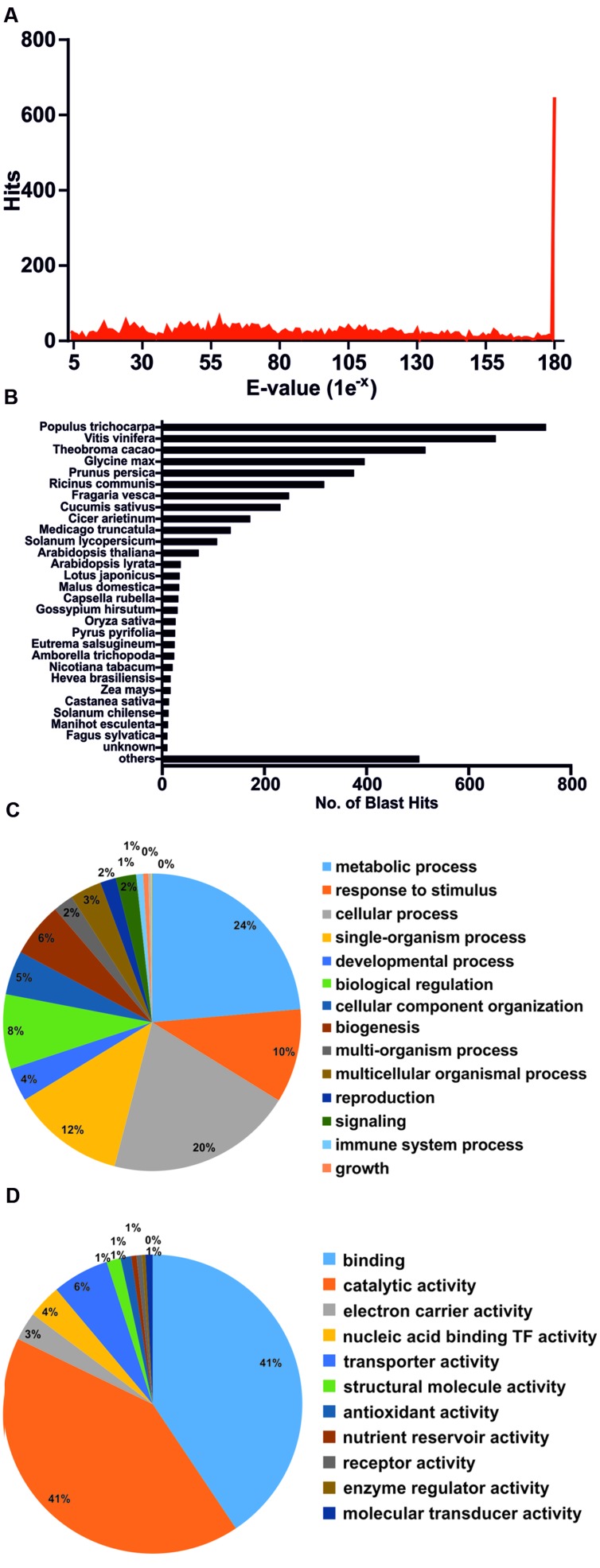
**Annotation of differentially expressed genes by BLAST homology search. (A)** *E*-value distribution of the first 50 BLAST hits per each unigene. **(B)** Species distribution of the first 50 BLAST hits per each unigene. **(C)** Functional categorization of annotated genes by Blast2GO (*GO Biological Process*) analysis. **(D)** Functional categorization of annotated genes by Blast2GO (*GO Molecular Function*) analysis.

To functionally annotate DEGs, these were subjected to BLASTx homology search against the Non-redundant GenBank Protein Database, as well as Blast2GO analysis (Conesa et al., 2005). The complete set of annotated genes is compiled in **Supplementary File S2**. Quality of the sequencing/assembly strategy was validated by various forms. **Figure 5A** depicts that the majority of unigene homology hits had extremely low *E*-values. Only 5.7% of DEGs failed to annotate with known proteins, even when threshold *E*-values were raised up to 1e-1 (data not shown). Finally, species distribution for BLAST hits placed unigene homologs within plant species (**Figure 5B**), supporting the initial trimming step that eliminated foreign sequences and contaminants.

**Table 2** highlights the most significant DEGs based on functional categories that are of interest to our study. DEGs included an important number of effector proteins traditionally associated with drought responses, such as proteins with chaperone activity (dehydrins and LEA proteins) (Umezawa et al., 2006; Shao et al., 2008), solute transporters and biosynthetic genes of compatible osmolites (Wang et al., 2003), and proteins associated with cell wall remodeling (Tenhaken, 2014). DEGs also included regulatory components of known drought-related signaling pathways, such as TFs, Protein Phosphatase 2C (PP2Cs) (Schweighofer et al., 2004) and serine/threonine kinases (SnRKs) (Yoshida et al., 2002), as well as genes associated with ubiquitin-mediated protein degradation (Lee and Kim, 2011), suggesting a tight control of drought responses at both the transcriptional and protein turnover levels. Subsequently, Blast2GO was used to classify genes according to GO terms (**Figures 5C,D**). In support of our differential expression analysis, foremost *GO Biological Process* terms included metabolic processes and response to stimulus, while significant *GO Molecular Function* terms included nucleic acid binding TF activity and transporter activity. Interestingly, a series of genes normally considered to have housekeeping functions could be observed, including ribosomal-, actin-, and tubulin-gene family members (**Supplementary File S2**). Since genes belonging to these categories have already been used as constitutive expressors in *Q. suber* (Marum et al., 2012; Rocheta et al., 2014; Sebastiana et al., 2014; Teixeira et al., 2014), the present and ongoing NGS-based transcriptional studies will be important for a future revision of constitutive expressor genes.

**Table 2:** Selection and categorization of significant *Quercus suber* unigenes differentially expressed in response to drought.

Unigene ID	DEG	*Arabidopsis* AGI code	Gene name	Annotation
**Signaling**				
QSDrought_00285	Up	AT4G30960	*CIPK6*	ATCIPK6 CIPK6 SIP3 SNRK3.14
QSDrought_03501	Up	AT5G55090	*MAPKKK15*	MAPKKK15 mitogen-activated protein kinase kinase kinase 15
QSDrought_03551	Up	AT4G33950	*ATOST1*	ATOST1 SNRK2.6 SRK2E Protein kinase superfamily protein
QSDrought_04452	Up	AT5G10930	*SnRK3.24*	CIPK5 SnRK3.24 CBL-interacting protein kinase 5
QSDrought_06388	Down	AT5G50860	*AT5G50860*	Protein kinase superfamily protein
QSDrought_06651	Up	AT2G48010	*RKF3*	RKF3 receptor-like kinase in in flowers 3
QSDrought_06886	Up	AT3G25250	*AGC2*	AGC2 AtOXI1 AGC (cAMP-dependent, cGMP-dependent and protein kinase C) kinase family protein
QSDrought_08928	Up	AT5G47070	*AT5G47070*	Protein kinase superfamily protein
QSDrought_09509	Up	AT3G45640	*ATMPK3*	ATMAPK3 ATMPK3 MPK3 mitogen-activated protein kinase 3
QSDrought_09888	Down	AT1G11330	*AT1G11330*	*S*-locus lectin protein kinase family protein
QSDrought_11117	Down	AT2G21480	*AT2G21480*	Malectin/receptor-like protein kinase family protein
QSDrought_14166	Up	AT2G33580	*AT2G33580*	Protein kinase superfamily protein
QSDrought_20555	Up	AT5G60550	*GRIK2*	GRIK2 geminivirus rep interacting kinase 2
**Protein serine/threonine phosphate activity**				
QSDrought_01105	Up	AT1G07430	*HAI2*	HAI2 highly ABA-induced PP2C gene 2
QSDrought_03606	Up	AT2G29380	*HAI3*	HAI3 highly ABA-induced PP2C gene 2
QSDrought_06016	Up	AT3G62260	*AT3G62260*	Protein phosphatase 2C family protein
QSDrought_07848	Up	AT1G72770	*HAB1*	HAB1 homology to ABI1
QSDrought_09366	Up	AT2G30020	*AP2C1*	AP2C1 clade B of the PP2C-superfamily
QSDrought_11908	Up	AT1G34750	*AT1G34750*	Protein phosphatase 2C family protein
QSDrought_12112	Up	AT5G59220	*HAI1*	HAI1 SAG113 highly ABA-induced PP2C gene 1
**LEA**				
QSDrought_09652	Up	AT5G06760	*LEA4-5*	Late embryogenesis abundant 4–5
QSDrought_06522	Up	AT2G35980	*NHL10*	LEA hydroxyproline-rich glycoprotein 10
QSDrought_07106	Up	AT2G35981	*NHL10*	LEA hydroxyproline-rich glycoprotein 10
QSDrought_08053	Up	AT1G52690	*LEA7*	Late embryogenesis abundant 7
**Chaperone activity**				
QSDrought_16809	Up	AT3G03773	*HSP20-like*	HSP20-like chaperones superfamily protein
QSDrought_12322	Up	AT1G20450	*EDR10*	Dehydrin family protein ERD10
QSDrought_07218	Up	AT1G76180	*ERD14*	Dehydrin family protein ERD14
QSDrought_13469	Up	AT4G10710	*SPT16*	Global transcription factor C
**Cell wall remodeling**				
QSDrought_00248	Up	AT3G45970	*ATEXLA1*	Expansin-like A1
QSDrought_01805	Up	AT4G17030	*ATEXLB1*	Expansin-like B1
QSDrought_05775	Up	AT4G38400	*ATEXLA2*	Expansin-like A2
QSDrought_11848	Up	AT4G17030	*ATEXLB1*	Expansin-like B1
QSDrought_06025	Down	AT4G14130	*XTH15*	Xyloglucan endotransglucosylase/hydrolase 15
QSDrought_05100	Up	AT4G25810	*XTH23*	Xyloglucan endotransglycosylase 6
QSDrought_07044	Up	AT5G57550	*XTH25*	Xyloglucan endotransglucosylase/hydrolase 25
**Sugar:hydrogen symporter activity**				
QSDrought_00469	Up	AT2G43330	*ATINT1*	ATINT1 INT1 inositol transporter 1
QSDrought_00694	Up	AT3G18830	*ATPLT5*	ATPLT5 ATPMT5 PMT5 polyol/monosaccharide transporter 5
QSDrought_01264	Down	AT1G11260	*STP1*	ATSTP1 STP1 sugar transporter 1
QSDrought_02773	Up	AT1G30220	*ATINT2*	ATINT2 INT2 inositol transporter 2
QSDrought_04922	Up	AT5G26340	*MSS1*	ATSTP13 MSS1 STP13 Major facilitator superfamily protein
QSDrought_07023	Down	AT1G22710	*SUC2*	ATSUC2 SUC2 SUT1 sucrose-proton symporter 2

### Functional Data Mining of the Differential Transcriptome

**FIGURE 6:**
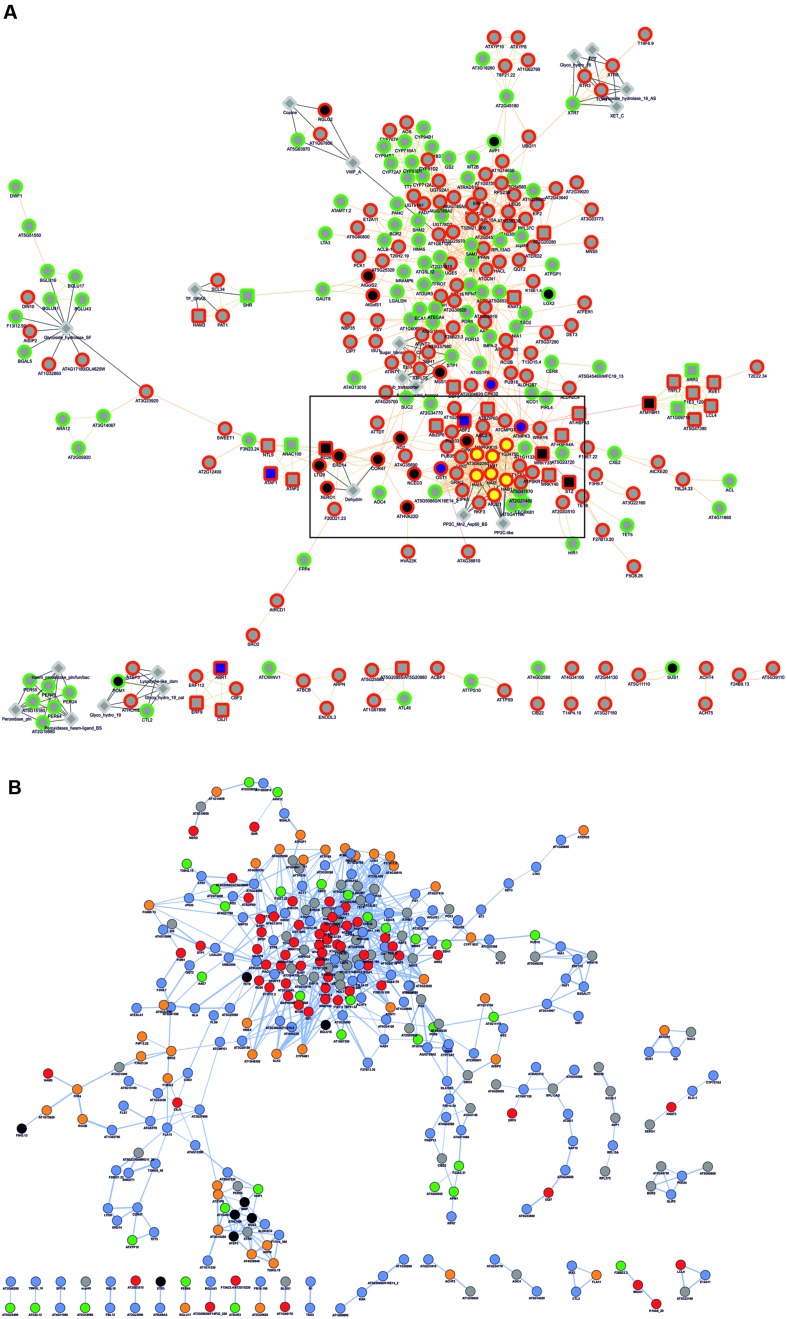
**Gene network analysis of *Arabidopsis* DEG orthologs. (A)** Analysis regarding functional features (attributes, genetic interactions, physical interactions, predicted protein interactions and shared protein domains), performed in the Genemania App of Cytoscape. Genes are generally represented as gray circles; attributes are represented as gray diamonds; genes automatically annotated as belonging to the GO category *Response to water* are represented as black nodes, and within these, those belonging to ABA-related subcategories are represented by blue nodes; genes automatically annotated as belonging to the GO category *Protein serine/threonine phosphatase activity* are represented as yellow nodes. Borders indicate up-regulated (red) and down-regulated (green) genes. Interactions are represented as edges (edge thickness represents normalized link weights) and edge color indicates physical interaction (magenta), predicted interaction (orange), shared protein domain (light green) and InterPro protein classification (gray). **(B)** Predicted protein co-localization analysis. Edge thickness represents normalized co-localization strength of the gene pair. Genes are represented by color code, reflecting predicted sub-cellular targeting of their coding proteins, based on GO *Cellular Component* categorization toward the nucleus (red), cytoplasm (blue), plasma membrane (orange), organelle (green), extracellular region (black) and unknown (gray).

To obtain functional data from *Q. suber* DEGs, we used a homology-based approach to identify DEG orthologs in the model plant *A. thaliana*, using BLASTp homology search against the *Arabidopsis* TAIR 10 genome annotation (**Figure 2**). Of the initial 546 *Q. suber* unigenes, 41 failed to annotate to any known sequence, and 15 did not annotate to *Arabidopsis* protein models, resulting in 490 BLAST hits. We also observed redundancy within these hits, leading to the identification of 454 unique DEG orthologs (**Figure 2**, **Supplementary File S2**). Functional data mining was subsequently performed by means of gene network analysis, using the Genemania plugin at Cytoscape (Warde-Farley et al., 2010; Saito et al., 2012). First, a functional network was created to represent known genetic interactions, protein interactions and protein structural features (**Figure 6A**). We used the built-in feature of Genemania to calculate and display the most over-represented GO categories of our dataset, uncovering a central gene cluster that was enriched in water responsive genes (the most over-represented GO category in the Genemania analysis), including various genes related to ABA-signaling categories, such as phosphatases of the PP2C family that are key regulatory components of ABA-signaling pathways, and other low Ψ_w_ responsive genes (Supplementary Figure S4A) (Saez et al., 2004; Bhaskara et al., 2012; Zhang et al., 2013).

A second network was generated to represent cellular co-localization (**Figure 6B**). Analysis revealed a major gene cluster associated with protein targeting to the nucleus, suggesting once more the existence of extensive transcriptional regulatory events. Based on this evidence, and considering that genes with identical expression patterns will be controlled by the same TFs and share common *cis*-elements in their promoters, we identified statistically over-represented *cis*-elements in the promoters of DEG orthologs (**Table 3**). We could observe that a significant number of over-represented TF-binding site motifs belonged to the conserved ABA-responsive *cis*-element (*ABRE*), and *ABRE*-like motifs. These elements are the binding site of *ABRE-binding factor* (ABF) TFs that have been previously associated to promoter regions of ABA-regulated, drought-responsive genes (Yoshida et al., 2010). All DEG orthologs that displayed *ABRE* and/or *ABRE*-like motifs were considered for co-expression network prediction using Cytoscape, and as expected, a complex gene co-expression network could be observed, indicating the existence of an ABA-mediated ABF-dependent regulon (Supplementary Figure S4B).

**Table 3:** *Cis*-elements over-represented in the promoter region of DEG orthologs.

*Cis* Element	Motif	*p*-Value (AtCOECis)	*p*-Value (Athena)
**Up-regulated genes**			
ABFs binding site motif	CACGTGGC	2.17e-3	<10e-4
ABRE binding site motif	BACGTGKM	2.57e-4	<10e-4
ABRE-like binding site motif	YACGTGGC	9.75e-5	<10e-10
ABREATRD22	RYACGTGGYR	3.22e-4	<10e-3
ACGTABREMOTIFA2OSEM	ACGTGKC		<10e-10
CACGTGMOTIF	CACGTG		<10e-7
DRE core motif	GACCGACTA	1.80e-3	<10e-4
DREB1A/CBF3	RCCGACNT		<10e-4
G-box plus G	CACGTGG	1.30e-5	
GADOWNAT	ACGTGTC		<10e-8
GAREAT	TAACAAR		<10e-3
GBOXLERBCS	MCACGTGGC		<10e-3
LRE	ACGTGGCA	7.00e-4	
NCS-motif	Various	1.52e-7	
TATA-box Motif	TATAAA		<10e-4
TGA1 binding site motif	TGACGTGG		<10e-3
UPRMOTIFIAT	CCACGTCA		<10e-3
**Down-regulated genes**			
MYB binding site promoter	CACCTAAC	1.09e-3	
NCS-motif	Various	<2,64e-6	

*The subset of up- and down-regulated genes were submitted to AtCOECis and Athena databases*.

Next we performed an automated search for TFs within DEG orthologs, and observed that drought imposition transcriptionally regulated a large variety of TFs (**Table 4**). The most abundant classes included NAC, AP2/ERF, WRKY and MYB TFs. The existence of various TF classes in the differential transcriptome of *Q. suber* roots responding to drought is extremely noteworthy. NAC, AP2/ERF, WRKY and MYB TFs have all been functionally associated with drought stress response mechanisms (Wang et al., 2003; Mahajan and Tuteja, 2005; Shao et al., 2008). For instance, in *A. thaliana*, loss- or gain-of-function mutants for *ATAF1* (Lu et al., 2007), *MYB44* (Persak and Pitzschke, 2014), *ABR1* (Pandey et al., 2005) and *WRKY40* (Chen et al., 2010; Wang et al., 2013) have been established as drought/osmotic stress determinants, regulating signaling pathways, ROS scavenging, cell wall remodeling and stomatal aperture. In order to validate BLAST homology searches and the existence of drought-related *Q. suber* TFs, these were phylogenetically resolved against the totality of annotated *Arabidopsis* TFs, in each of the four most represented TF classes (Supplementary Figure S5). Results allowed us to identify the closest *Arabidopsis* orthologs to our *Q. suber* genes-of-interest, which is important for two reasons. On the one hand, identification of *Arabidopsis* orthologs with known functional association with drought resistance can be used to functionally validate *Q. suber* genes as novel cork oak determinants of root level drought stress resistance, namely by complementation of *Arabidopsis* functional mutants with *Q. suber* orthologs. Secondly, phylogenetic mapping of *Q. suber* drought-responsive genes can be used in exploratory analysis to identify novel drought stress determinants in *Arabidopsis*, which can then be validated by subsequent functional mutant approaches in this model species. For instance, QSDrought_01793, a MYB TF, is the ortholog of At5g67300 (MYB44) and is likely to be its functional equivalent: MYB44 is a known determinant of salt and drought stress responses. On the other hand, within the same MYB TF family, QSDrought_16911 suggests that the functionally unresolved *Arabidopsis* At1g26580 gene is possibly a drought stress determinant.

**Table 4:** List of transcription factors (TFs) present within DEG orthologs.

TF family name	AGI code	Gene name, synonym	QS ID	Differential expression
Alfin-Like	AT5G20510	*AL5*	QSDrought_11056	Down-regulated
AP2-EREBP	AT2G33710		QSDrought_04959	Up-regulated
	AT3G50260	*ATERF#011, CEJ1, RAP2.1*	QSDrought_10869	Up-regulated
	AT4G25470	*CBF2, DREB1C, FTQ4*	QSDrought_05446	Up-regulated
	AT5G44210	*ERF9*	QSDrought_09244	Up-regulated
	AT5G64750	*ABR1*	QSDrought_13237	Up-regulated
ARR-B	AT4G16110	*ARP5, ARR2*	QSDrought_08994	Down-regulated
bHLH	AT1G35460		QSDrought_18928	Down-regulated
bZIP	AT1G42990	*bZIP60*	QSDrought_12067	Up-regulated
	AT1G45249	*ABF2, AREB1*	QSDrought_02295	Up-regulated
	AT2G22850	*bZIP6*	QSDrought_06726	Up-regulated
	AT2G46270	*GBF3*	QSDrought_20020	Up-regulated
C2H2	AT1G10480	*ZFP5*	QSDrought_06423	Up-regulated
	AT1G27730	*STZ, ZAT10*	QSDrought_01447	Up-regulated
	AT2G27100	*SE*	QSDrought_07992	Up-regulated
C3H	AT5G20885		QSDrought_17648	Up-regulated
GRAS	AT4G00150	*SCL6*	QSDrought_09538	Up-regulated
	AT5G48150	*PAT1*	QSDrought_16917	Up-regulated
	AT4G37650	*SGR7, SHR*	QSDrought_03719	Down-regulated
Homeobox	AT5G25220	*KNAT3*	QSDrought_04401	Up-regulated
HSF	AT4G18880	*HSFA4A, HSF21*	QSDrought_06244	Up-regulated
	AT5G03720	*HSFA3*	QSDrought_07727	Up-regulated
MYB	AT5G67300	*MYB44, MYBR1*	QSDrought_01793	Up-regulated
	AT4G12350	*MYB42*	QSDrought_06966	Up-regulated
	AT1G26580		QSDrought_16911	Up-regulated
	AT3G60460	*DUO1*	QSDrought_17304	Down-regulated
MYB-Like	AT4G01280		QSDrought_13589	Up-regulated
	AT5G04760		QSDrought_07946	Up-regulated
	AT5G17300		QSDrought_03809	Up-regulated
	AT5G47390		QSDrought_12736	Up-regulated
NAC	AT1G01720	*ANAC002, ATAF1*	QSDrought_04760	Up-regulated
	AT4G27410	*ANAC072, RD26*	QSDrought_03374	Up-regulated
	AT4G35580	*NTL9*	QSDrought_00747	Up-regulated
	AT5G08790	*ANAC081, ATAF2*	QSDrought_06066	Up-regulated
	AT5G61430	*ANAC100, ATNAC5*	QSDrought_06406	Down-regulated
RAV	AT1G13260	*RAV1*	QSDrought_14492	Up-regulated
WRKY	AT1G62300	*WRKY6*	QSDrought_05433	Up-regulated
	AT1G80840	*WRKY40*	QSDrought_03742	Up-regulated
	AT2G38470	*WRKY33*	QSDrought_06729	Up-regulated
ZF(CCCH-type)	AT2G20280		QSDrought_12281	Up-regulated

*For identification purposes, genes were submitted to the AGRIS and MapMan platforms.*

### Identification of a Core ABA-Dependent Signaling Network

**FIGURE 7:**
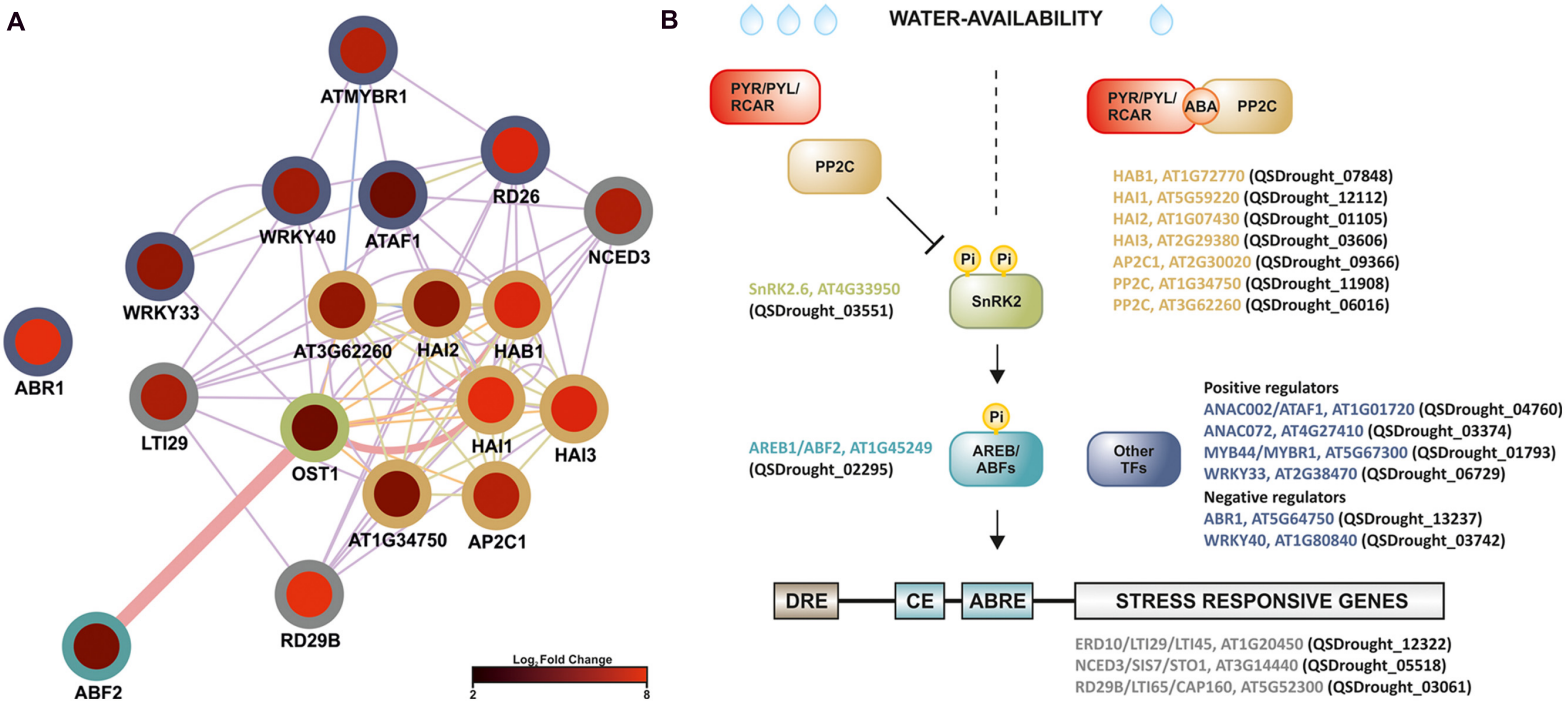
**Model for the regulation of long-term drought responses in *Q. suber* roots, where *ABF*-dependent signaling pathway components are up-regulated in roots exposed to long-term drought. (A)** Functional network (functional features on Cytoscape), based on known *Arabidopsis* ABF-dependent signaling components (Miyakawa et al., 2013; Nakashima and Yamaguchi-Shinozaki, 2013), cross-referenced against the complete DEG functional network. Edge thickness represents normalized link weights and edge color indicates physical interaction (magenta), predicted interaction (orange), shared protein domain (light green) and co-expression (purple). Node color represents expression in Log_2_ of fold change, while border color represents different classes of signaling components. **(B)** Schematic model of the proposed ABF-dependent signaling cascade: colors refer to different *Arabidopsis* signaling cascade components (gene name plus AGI code), while brackets highlight matching Quercus ID unigenes, found in the present analysis to be differentially expressed. Low water availability induces ABA production, which is recognized by the PYR/PYL/RCAR receptors. PYR/PYL/RCAR’s structural conformation changes after binding to ABA, thus recruiting Protein Phosphatase 2C (PP2C) phosphatases. PP2Cs are unavailable to dephosphorylate SnRK2 kinases. Phosphorylated (active) SnRK2s activate AREB/ABF transcription factors (TFs), which recognize *ABRE cis*-element motifs, mediating induction of stress-related gene expression (e.g., *RD29b* and *NCED3*). Other TF families are also likely to play a role in drought-signaling, including ANAC, AP2/EREB, MYB and WRKY TFs.

In plants, drought stress responses have been functionally mapped to four major signaling pathways that involve both ABA-dependent and ABA-independent pathways (Shinozaki and Yamaguchi-Shinozaki, 2007; Kuromori et al., 2014). Present results strongly supported the involvement of an ABA-dependent signaling network in the *Q. suber* root-level response to drought, given that (1) analysis of GO category enrichment highlighted the up-regulation of known ABA-signaling components, (2) *cis*-element analysis proposed an enrichment in *ABRE* and *ABRE-like* motifs, (3) several ABA-related TFs and response marker genes were identified. Present evidence specifically suggested the existence of ABF-related signaling events, therefore we performed an intersection network, merging the DEG functional network and components of the ABA- and ABF-dependent signal transduction pathway (**Figure 7A**). Surprisingly, we uncovered evidence for the transcriptional activation of the complete core ABA signal transduction pathway in long-term drought-stressed *Q. suber* roots. ABA levels can be sensed by a series of different cellular receptors (Golldack et al., 2014). In the best established case, PYR/PYL/RCAR nucleocytoplasmic receptors bind ABA and inhibit PP2Cs, preventing them from negatively regulating SnRK2 kinases. SnRK2s are then free to phosphorylate and activate key components that include ABF TFs, thus modulating the activity of a series of ABA-responsive genes (**Figure 7B**) (Raghavendra et al., 2010; Nakashima and Yamaguchi-Shinozaki, 2013; Golldack et al., 2014; Kuromori et al., 2014). Here we observed that, with the exception of the PYR/PYL/RCAR sensor, all components could be found within *Q. suber* DEGs, and all appeared to be up-regulated upon stress imposition. One *Q. suber PYR*/*PYL*/*RCAR* gene was found in the assembled *Q. suber* transcriptome, suggesting that the sensor is constitutively expressed.

The most observed enrichment related to the *PP2C* gene family, with seven up-regulated genes (**Table 2**, **Figure 7B**). *Q. suber PP2C* were phylogenetically resolved against the *Arabidopsis PP2C* gene family (Supplementary Figure S6). Components included orthologs of *Arabidopsis* HAB1, a negative regulator of the ABA signaling pathway (Saez et al., 2004), HAI1, also associated with negative regulation of ABA signaling pathway (Zhang et al., 2013), and HAI2 and HAI3, that together with HAI1 have been associated with negative regulation of osmotic adjustment-related genes (Bhaskara et al., 2012). Interestingly, ortholog PP2Cs also include AP2C1, reported as being both a negative regulator of MAPK signaling proteins, and a regulator of ABA-related genes and signaling components (Schweighofer et al., 2007; Brock et al., 2010). Positive components of the pathway (*SnRK2* and *ABF* elements) were also observed and found to be up-regulated, possibly contributing for the transcriptional accumulation of drought stress marker genes such as *RD29b* and *NCED3*, both of which respond to ABF by containing *ABRE cis*-elements (**Figure 7B**) (Nakashima et al., 2006; Behnam et al., 2013). NCED3 is a component of the ABA biosynthetic pathway (Iuchi et al., 2001), and may thus contribute to signal amplification via a positive feedback loop.

**FIGURE 8:**
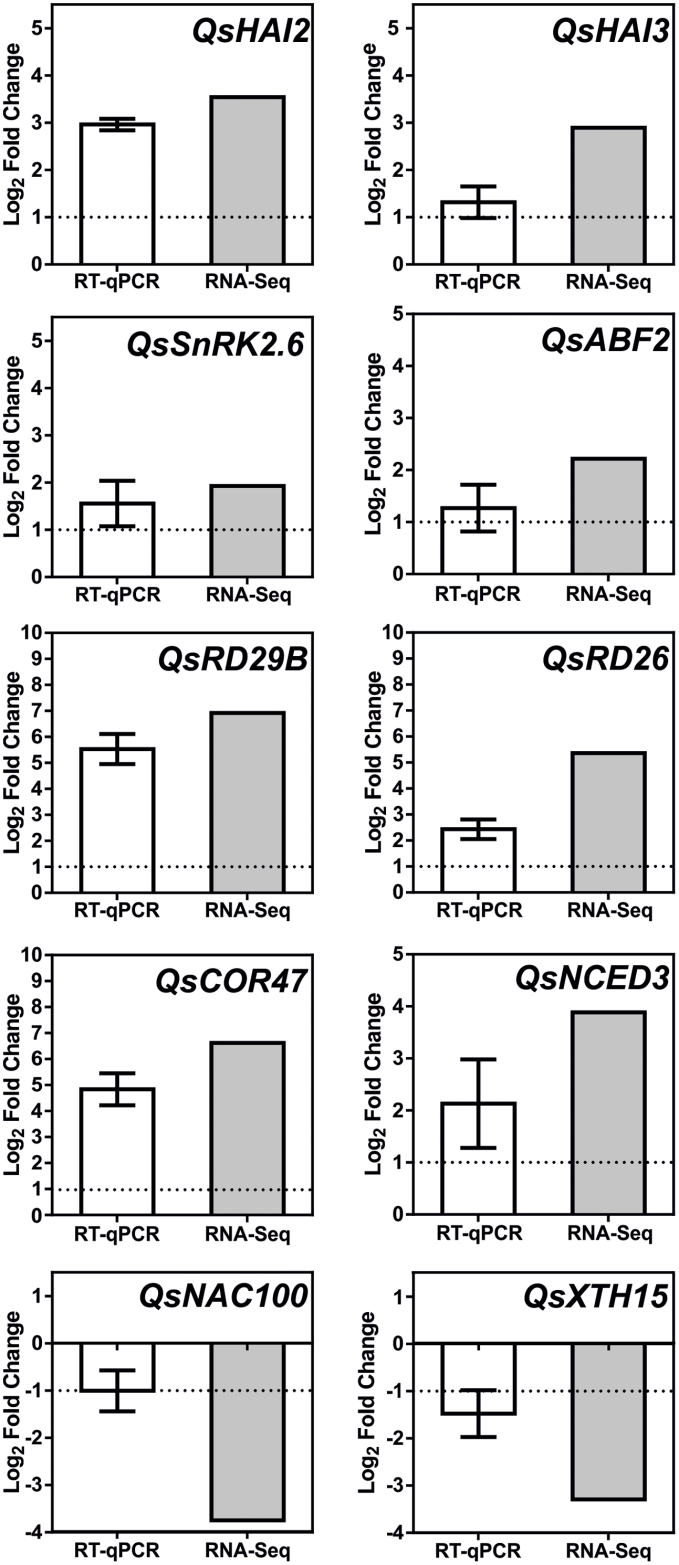
**Real-time PCR (qPCR) analysis of selected *Q. suber* drought stress responsive genes.** White bar represents changes in mRNA levels of root tissues under severe drought stress (D25/D10), in comparison to control samples (D100/90). Expression was normalized using *QsPP2A-3* as a reference gene. Results represent mean values ± SD (*n* = 3) and are expressed as Log_2_ values of the fold change. Gray bar represents matching relative expression values, derived from RNA-Seq analysis of the D100/90 vs. D25/10 condition.

Additional signaling pathways may be involved in the *Q. suber* root response to long-term drought, given the extension of TFs and genes involved in the overall transcriptional response to drought, and the capacity of this organism to sustain long periods of water deficit. **Figure 7B** acknowledges the existence of additional regulatory elements of the *Q. suber* differential transcriptome, namely TFs that have been previously highlighted (**Table 4**), and that act as both positive and negative determinants of drought-related effector genes (Fujita et al., 2004; Pandey et al., 2005; Lu et al., 2007; Yoshida et al., 2010; Nakashima and Yamaguchi-Shinozaki, 2013; Wang et al., 2013; Persak and Pitzschke, 2014). To functionally validate our observations, qPCR was used to determine gene expression and compare it to RNA-Seq data (**Figure 8**). Emphasis was given to the above-mentioned ABA-dependent ABF signaling pathway. Therefore, genes selected for qPCR analysis incorporated at least one example of the components of the pathway, from *PP2C* to effector genes. All ten genes closely matched the relative expression values calculated via RNA-Seq analysis, thus supporting the current experimental strategy.

## Conclusion

As environmental changes in the Mediterranean basin keep raising concern on the future resilience of *Q. suber*, and as numbers of cork oak populations keep declining, more and more efforts are required to understand the biology and physiology of such an economically significant species. Over the last years, tools have been developed to study several aspects of cork oak biology at the genetic and molecular levels, such as gene/protein characterization, population genomics and dEST transcriptomics. However, the advent of an *Omics* age for this species is essential for the future characterization of cork oak at a systems level. A significant step will be accomplished with the ongoing sequencing of its full genome. In the present report, we used an RNA-Seq approach to uncover that, upon long-term drought stress imposition, *Q. suber* roots induce all known components of an ABA-dependent signaling network involving ABF TFs. This pathway seems to be part of an even larger transcriptional rearrangement that should be the subject of future studies, bringing new insight onto the cork oak response to low water availability. Moreover, the present report opens new research possibilities, as it can be used as an exploratory study towards the identification of novel stress determinants in the model species *A. thaliana*.

## Author Contributions

HA, RT, and TL-N designed and supervised the drought experiment; AM, NV, DC, FR, TL-N and HA grew plants, performed RNA extraction and physiological characterization; HA and PHC designed and supervised the bioinformatics and gene network analysis strategies; AM, IM, NV, and HA performed bioinformatics; AM, NV, FR, and PC performed qPCR analysis; All authors discussed the data. HA, RT, TL-N, PC, and AM discussed the data and wrote the manuscript.

## Conflict of Interest Statement

The authors declare that the research was conducted in the absence of any commercial or financial relationships that could be construed as a potential conflict of interest.

## Footnotes

1 http://www.uniprot.org/uploadlists/

2 http://www.fagaceae.org/

3 http://www.arabidopsis.org/

4 http://bio-bwa.sourceforge.net

5 http://bioinfogp.cnb.csic.es/tools/venny/index.html

6 http://cdbfasta.sourceforge.net/

7 http://www.tm4.org/mev.html

8 http://bioinformatics.ysu.edu/tools/OrfPredictor.html

9 http://www.arabidopsis.org

10 http://arabidopsis.med.ohio-state.edu/AtTFDB/

11 http://www.corkoakdb.org/

## Supplementary material

**FILE S1 | FASTA file of transcriptome sequences.**

**FILE S2 | Complete annotation of DEGs.**
