# Supplementary material for: RNA-Seq and Gene Network Analysis Uncover Activation of an ABA-Dependent Signalosome During the Cork Oak Root Response to Drought
Source: Front Plant Sci. 2016 Jan 11;6:1195. doi: 10.3389/fpls.2015.01195 (PMC4707443; doi:10.3389/fpls.2015.01195)
Supplement: Supplementary file 3 [file Supplementary_Data.DOCX]

Supplementary Material

RNA-Seq and gene network analysis uncover activation of an ABA-dependent signalosome during the cork oak root response to drought

Alexandre Papadopoulos Magalhães1; Nuno Verde1; Francisca Reis1; Inês Martins1; Daniela Costa1; Teresa Lino-Neto1; Pedro Humberto Castro1; Rui Manuel Tavares1,*; Herlânder Azevedo2,*

^1^ BioSystems & Integrative Sciences Institute (BioISI), Plant Functional Biology Center, University of Minho, Campus de Gualtar, 4710-057 Braga, Portugal

^2^ CIBIO, InBIO - Research Network in Biodiversity and Evolutionary Biology, Universidade do Porto, Campus Agrário de Vairão, 4485-661 Vairão, Portugal

**Correspondence:**

Herlânder Azevedo, CIBIO, InBIO - Research Network in Biodiversity and Evolutionary Biology, Universidade do Porto, Campus Agrário de Vairão, 4485-661 Vairão, Portugal

Email: hazevedo@cibio.up.pt

Rui Manuel Tavares, BioSystems & Integrative Sciences Institute (BioISI), Plant Functional Biology Center, University of Minho, Campus de Gualtar, 4710-057 Braga, Portugal

Email: tavares@bio.uminho.pt

# Supplementary Figures, Tables and Methods

The following supplementary figures, tables and methods are included:

**Supplementary Figure 1.** Characterization of sequence data.

**Supplementary Figure 2.** Homology between *Q. suber* transcriptomes.

**Supplementary Figure 3.** Characterization of differential gene expression.

**Supplementary Figure 4.** Additional gene network analysis.

**Supplementary Figure 5.** Phylogenetic reconstruction of the TF classes.

**Supplementary Figure 6.** Phylogenetic reconstruction of PP2C genes.

**Supplementary Table 1.** List of primers used in qPCR.

**Supplementary Methods 1.** Gene network analysis.

**Supplementary Methods 2.** Phylogenetic analysis.

**Supplementary Methods 3.** Quantitative Real-Time PCR.

## Supplementary Figures

**
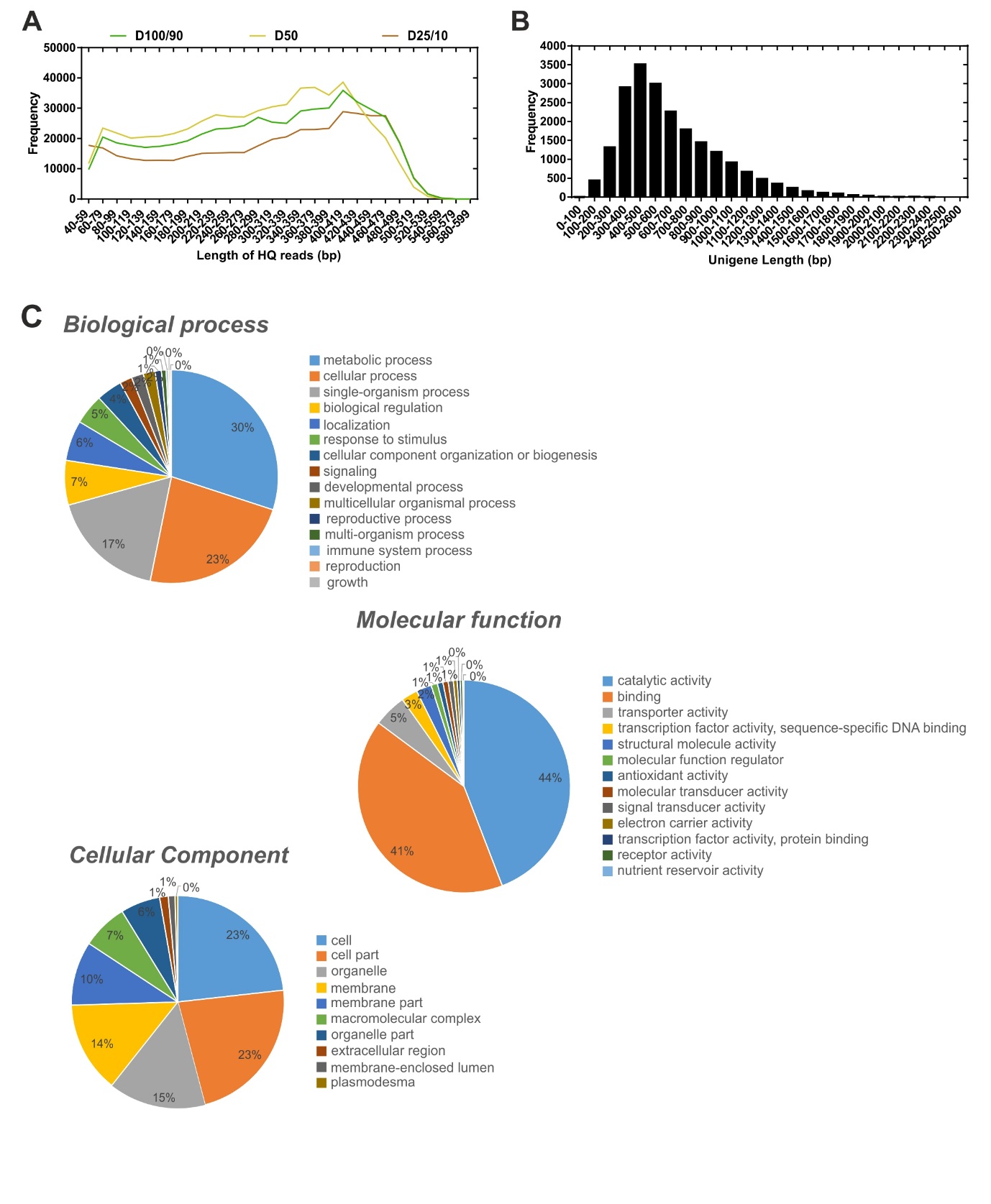
**

**Supplementary Figure 1. Characterization of sequence data regarding processing and assembly of reads.** **(A)** Distribution of the length of HQ reads obtained for each watering regime. **(B)** Distribution of the length of unigenes obtained after assembly. **(C)** GO term enrichment for the full assembled transcriptome.


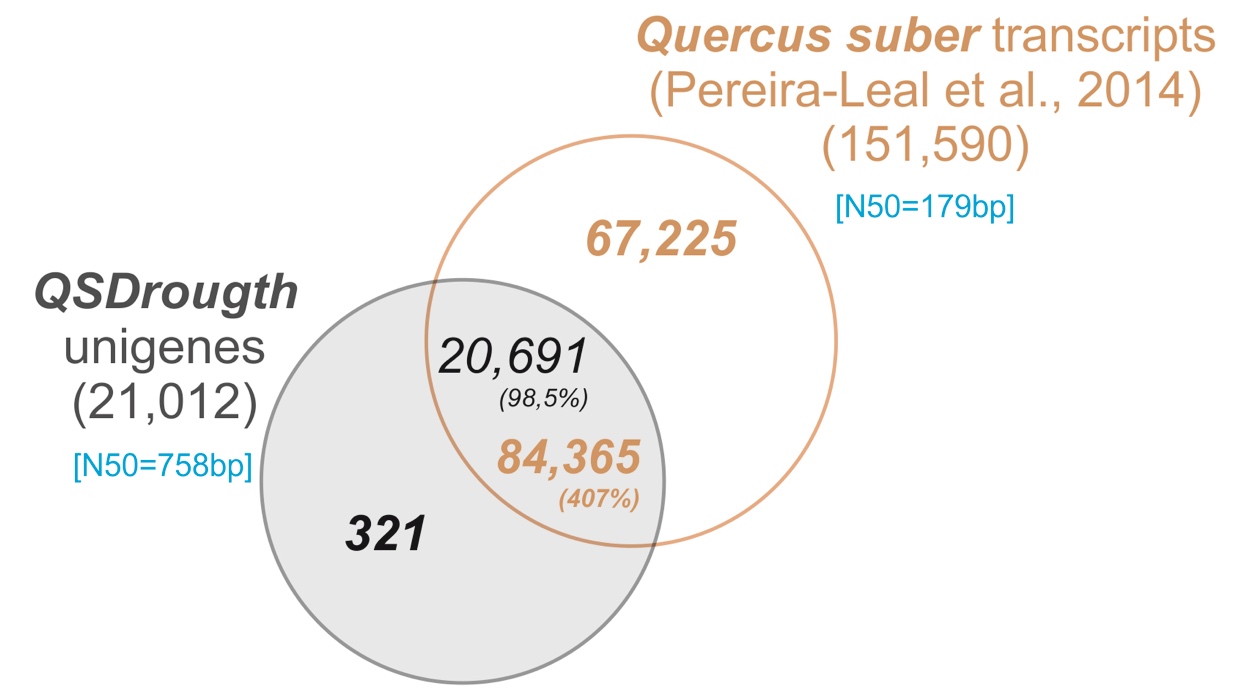


**Supplementary Figure 2**. Venn diagram representation of the number of BLAST homology hits (E-value<1e-6) between the currently assembled transcriptome, and the *Quercus suber* transcriptome by Pereira-Leal and co-workers (2014). Square brackets represent N50 values for transcript length.


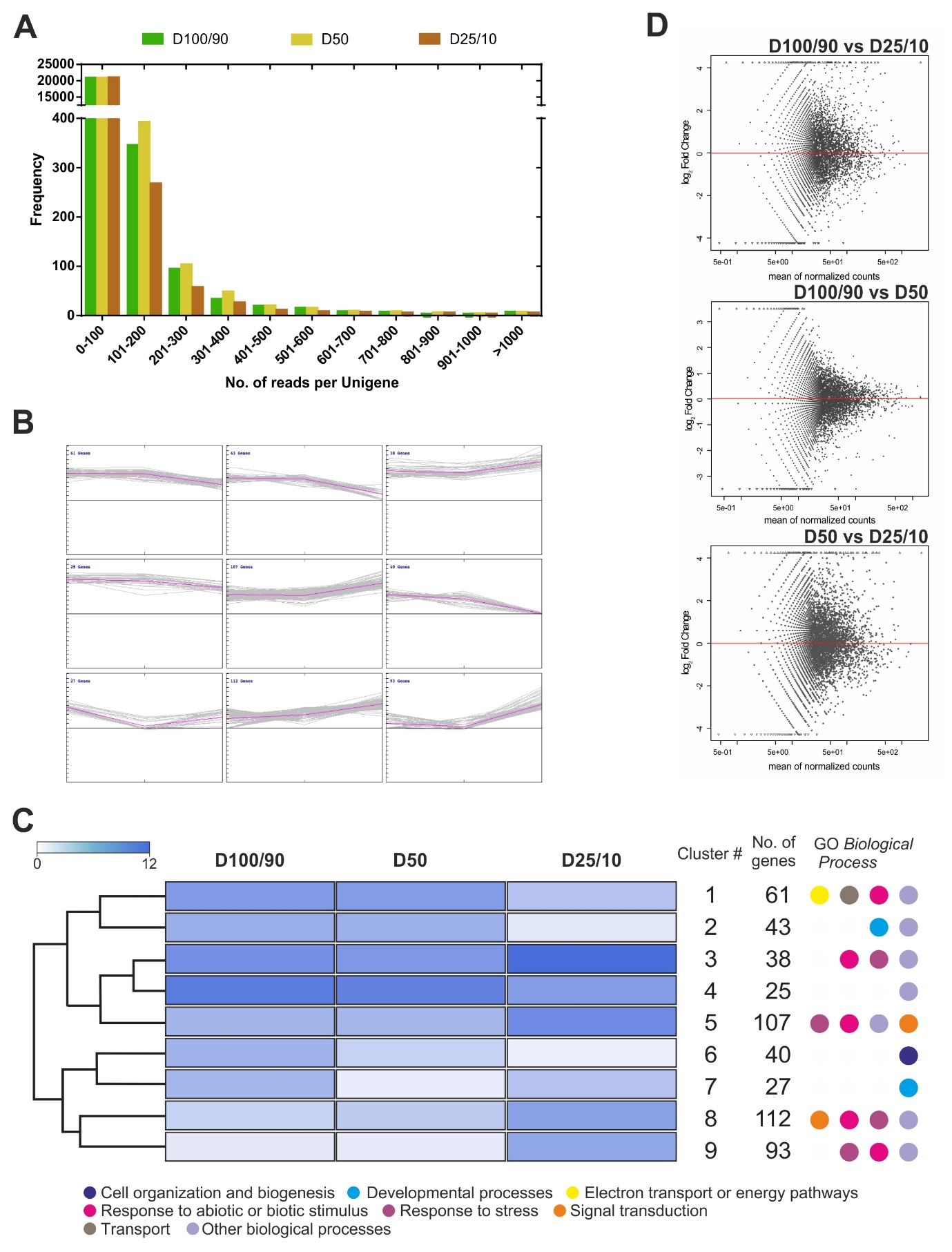


**Supplementary Figure 3. Characterization of differential gene expression in drought-stressed *Quercus suber* roots.** **(A)** Distribution, for each watering regime, of the number of reads mapped to each unigene (unigene coverage). **(B)** Expression plot of the 9 clusters identified by SOTA analysis. **(C)** Dendogram of the 9 clusters identified by SOTA analysis. Each gene cluster was submitted for GO enrichment analysis for *Biological process* (E-value=1e-5). Each enriched category is represented as a coloured dot, sorted by higher to lower significance from left to right. **(D)** MA plots of the paired differential expressions for D100/90 vs D50, D100/90 vs D25/10 and D50 vs D25/10. Graphs represent scatter plots of each individual unigene’s Log_2_ fold change in expression, as a function of the mean of normalized counts. Note that most of the genes are represented as having basal expression values (between zero and one-fold change) and few have large changes in expression, thereby validation DESeq parameters used for the differential expression analysis.


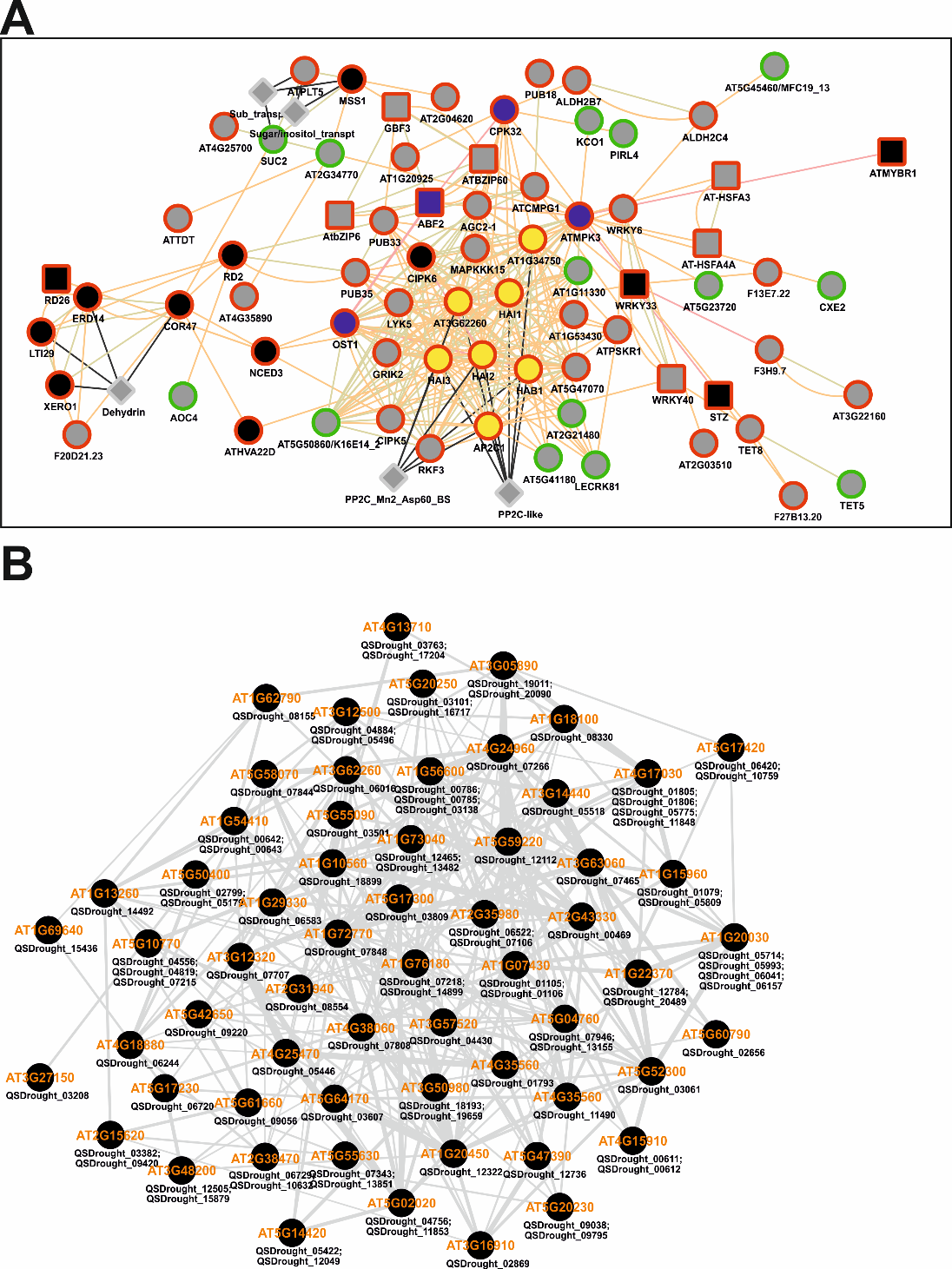


**Supplementary Figure 4. Additional gene network analysis, performed in the Genemania App of Cytoscape, of Arabidopsis DEG orthologs.** **(A)** Highlight of the main cluster of the functional feature analysis (Figure 6A). The core gene cluster is overrepresented in the three most enriched Gene Ontology co-annotation categories. Genes are represented as grey circles; attributes are represented as grey diamonds; genes automatically annotated as belonging to the GO category *Response to water* are represented as black circles, and within these, those belonging to ABA-related subcategories are represented by blue circles; genes automatically annotated as belonging to the GO category *Protein serine/threonine phosphatase activity* are represented as yellow circles. Borders indicate up-regulated (red) and down-regulated (green) genes. Interactions are represented as edges (edge thickness represents normalized link weights) and edge colour indicates physical interaction (magenta), predicted interaction (orange), shared protein domain (light green) and InterPro protein classification (grey). **(B)** Co-expression network of DEGs containing ABRE and ABRE-like motifs in their promoters. Genes are represented as black circles. Edge thickness represents normalized gene co-expression strength. Arabidopsis AGI gene codes are represented (orange), as well as their orthologs from the *Q. suber* DEG unigene set.


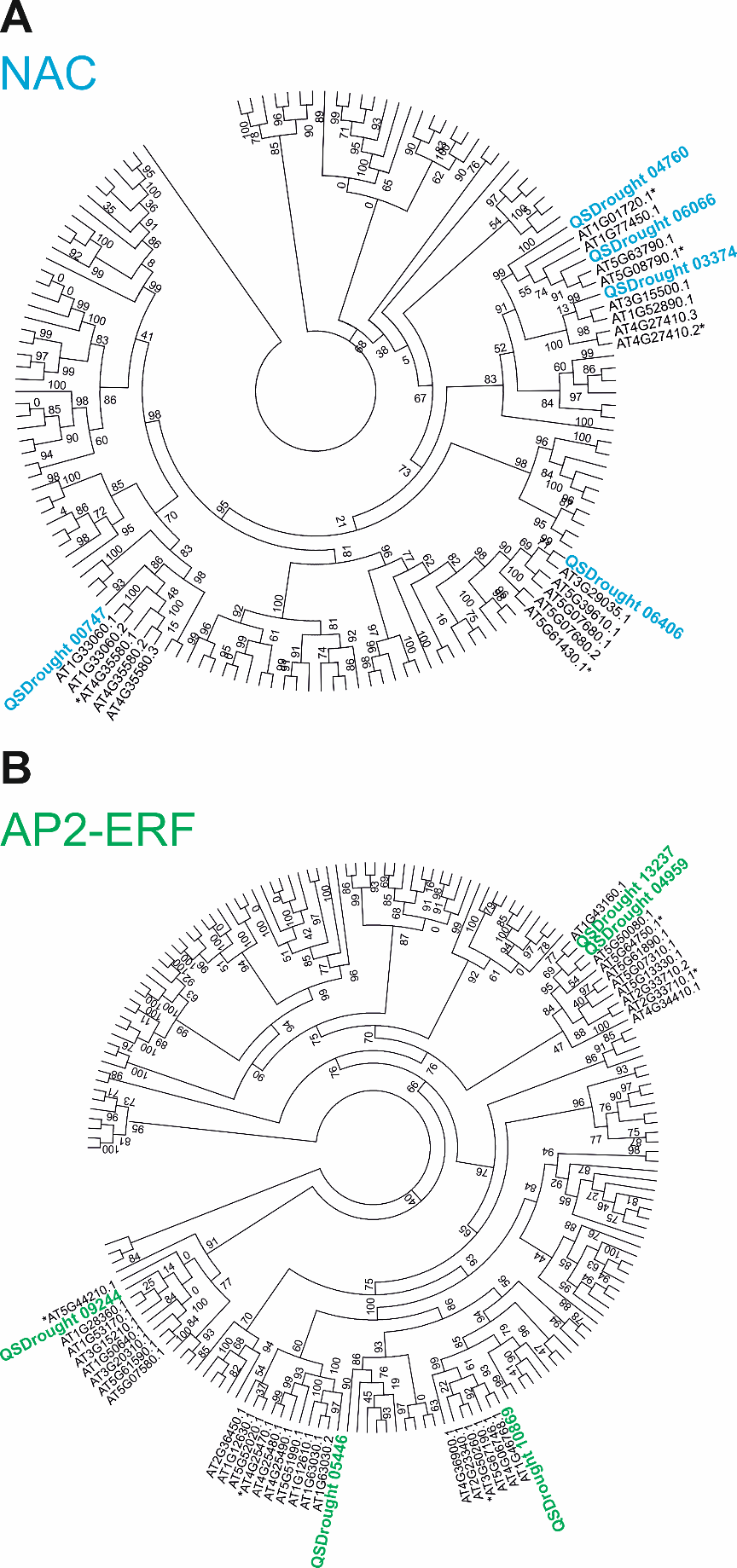


**
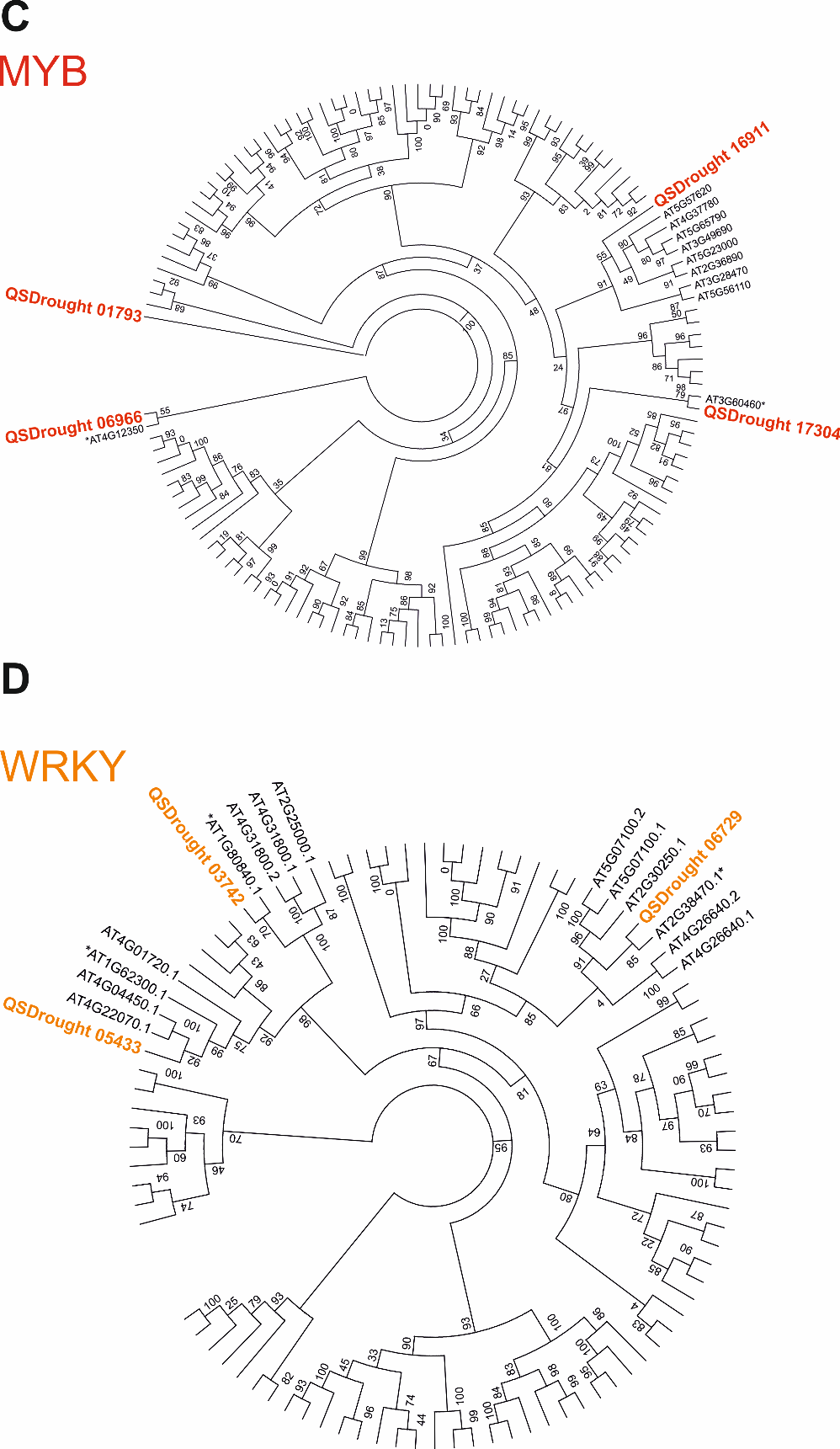
**

**Supplementary Figure 5. Phylogenetic reconstruction of the most overrepresented transcription factor classes present in the DEG list.** **(A)** NAC TF class phylogenetic reconstruction. **(B)** AP2-ERF TF class phylogenetic reconstruction. **(C)** MYB TF class phylogenetic reconstruction. **(D)** WRKY TF class phylogenetic reconstruction. All tress contain *Q. suber* differentially expressed unigenes, and the complete annotated set of *Arabidopsis thaliana* gene family members. The first BLAST hit from the ortholog identification step is marked with an asterisk**.**


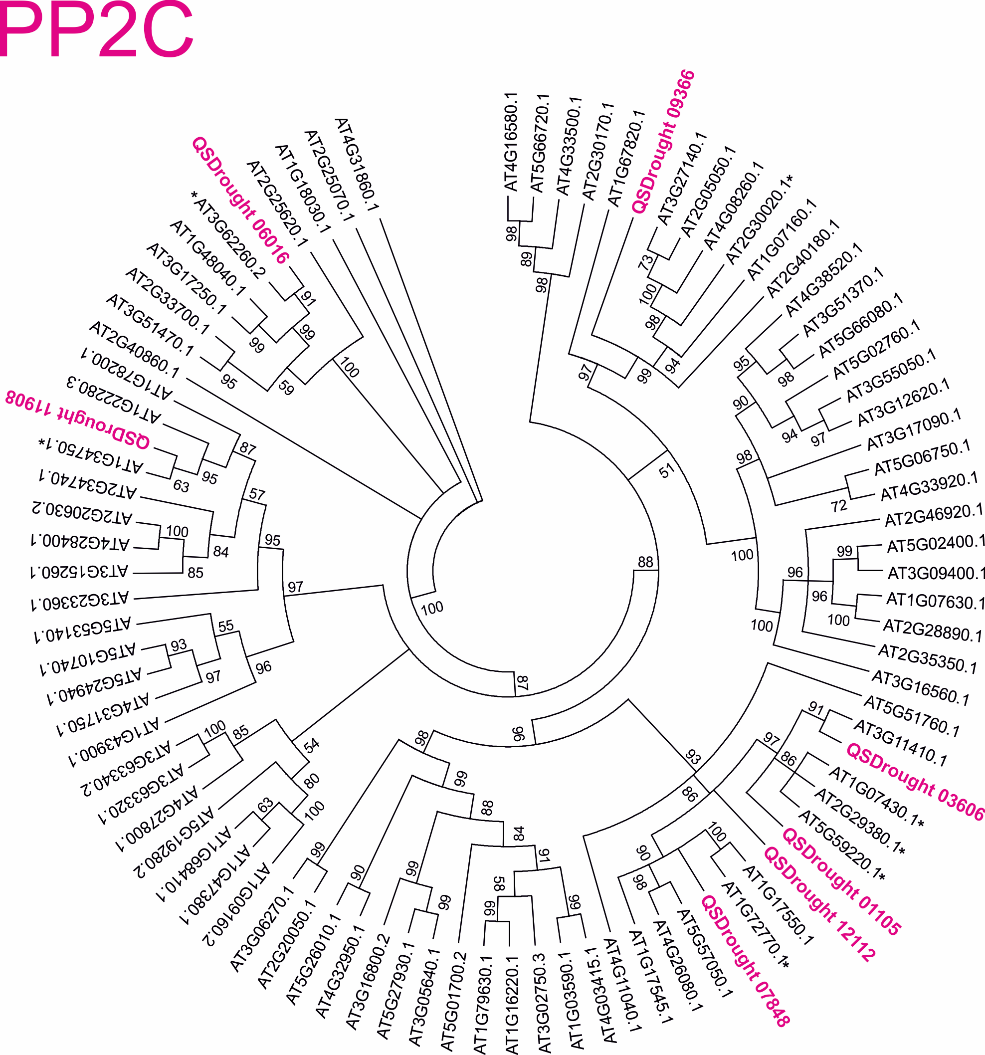


.

**Supplementary Figure 6. Phylogenetic reconstruction of PP2C.** Analysis includes unigenes identified in the *Q. suber* differential expression study, and the complete annotated set of *Arabidopsis thaliana* genes from the same class. Asterisks represent the first hit in the ortholog identification step.

## Supplementary Tables

**Supplementary Table 1. List of primers used in qPCR.**

| **Gene Name (Unigene ID)** | **Primer Sequence (5' to 3')** | **Length (bp)** | **Tm (ºC)** | **Product size (bp)** |
| --- | --- | --- | --- | --- |
| QsHAI2 | CAGGTGGTCGGGTCATCTAC | 20 | 60.4 | 181 |
| (QSDrought_01106) | ATCACGTCCCAAAGCCCATC | 20 | 59.5 |  |
| QsHAI3 | GTTCTGGGAATTTCGACTGGG | 21 | 60.1 | 176 |
| (QSDrought_03606) | CCACTCTGAACCTCGTCATCC | 21 | 58.9 |  |
| QsSNRK2.6 | AGCGGAATGAAGAGCCAGAG | 20 | 60.6 | 170 |
| (QSDrought_03551) | GAGCCTGTCCACCACACTTC | 20 | 59.8 |  |
| QsABF2 | TCGGATCCAAGAAGCTGGTG | 20 | 60.9 | 165 |
| (QSDrought_02295) | CGCTCGCCAGGATTAGACAC | 20 | 59.8 |  |
| QsRD29B | GCCAAGAGGATCACACTCCC | 20 | 59.8 | 167 |
| (QSDrought_03061) | TCTCCTGGCATCTTTGCCTC | 20 | 60.1 |  |
| QsRD26 | ACAGCCACTCTTCCCCAAAC | 20 | 59.8 | 175 |
| (QSDrought_03374) | CCTACCCTGCCAATTCCTCC | 20 | 60.2 |  |
| QsCOR47 | GTAGATGAACATGGGCCCCG | 20 | 60.3 | 145 |
| (QSDrought_05686) | CACCGCCATGAGTAGGTACG | 20 | 60.5 |  |
| QsNCED3 | TGCATGACTCCAGCTGACTC | 20 | 60.3 | 176 |
| (QSDrought_05518) | TTCCTTCCAAGCCTGTTCCG | 20 | 59.8 |  |
| QsXTH15 | CCCTTCCCTAAGAGCCAACC | 22 | 59.9 | 179 |
| (QSDrought_06025) | TGCCATGTAGAATCGGAGGAAG | 20 | 59.7 |  |
| QsNAC100 | AAGGGTAGAGCCCCAAAAGG | 22 | 58.1 | 111 |
| (QSDrought_06406) | CAGTCTCCAGTCTCACTATCCC | 20 | 60.1 |  |
| QsPP2A-3 | GGGTTCCCAACATCAAGTTC | 20 | 56.6 | 174 |
| (QSDrought_03365) | TGACCTGATCACTTGACTGC | 20 | 57.5 |  |

## Supplementary Methods

**Supplementary Methods 1. Gene network analysis.**

Gene networks were predicted in Cytoscape, using the Genemania plugin. Network layouts were as follows: functional features, Prefuse Force Directed Layout; sub-cellular localization, Edge-weighted Spring Embedded Layout; co-expression, Prefuse Force Directed Layout. Default parameters were used but changes were made so that only the DEG list was considered for analysis. For functional network prediction, Gene Ontology co-annotation was used to highlight clusters or genes of interest. For the co-localization network, GO ontology annotation of DEG orthologs was carried by the AgBase database web server (<http://agbase.msstate.edu/>). Genes were mapped for the second GO slim level and given distinct colours within the predicted network.

**Supplementary Methods 2. Phylogenetic analysis.**

For each class of transcription factors (TFs), we used automated gene family annotation to retrieve the a.a. sequences of all family members present in the *Arabidopsis thaliana* genome. For that purpose the Plaza web-based platform (version 2.5; Van Bel et al. 2012) was used. Phylogenetic analysis was performed in SeaView v4.5. (Gouy et al. 2010). Sequences of *Q. suber* differentially expressed family members and Arabidopsis family members were aligned with the MUSCLE algorithm (Edgar 2004). The PhyML v3.1 (Guindon et al. 2010) feature of Seaview was used to compute maximum likelihood trees using the LG substitution model (Le and Gascuel 2008), with branch support using the aLRT SH-like method (Anisimova and Gascuel 2006).

**Supplementary Methods 3. Quantitative Real-Time PCR.**

RNA quantity and quality were assessed using a Nanodrop ND-1000 spectrophotometer and electrophoretic analysis. RNA samples were treated with Recombinant DNase I (Takara Biotechnology), and cDNA was subsequently generated using a Superscript III Reverse Transcriptase Kit (Invitrogen). For qPCR reactions, Ssofast Evagreen Supermix (BIORAD) was used according to the manufacturer’s indications. Reactions used 1:20 cDNA dilutions. Analysis was run in a CFX96 Touch Real-Time PCR Detection System (BIORAD), as per the manufacturer’s instructions. Relative expression was determined by the Pfaffl equation (Pfaffl, 2001), normalizing with PP2A gene. Values were calculated between severe drought conditions (D25/10) and well-watered conditions (D100/90). Primers for qPCR were designed using NCBI Primer-BLAST (Ye et al. 2012).

## Supplementary References

Anisimova, M., and Gascuel, O. (2006). Approximate likelihood-ratio test for branches: A fast, accurate, and powerful alternative*. Syst. Biol*. 55**,** 539-552. doi:10.1080/10635150600755453

Edgar, R.C. (2004). MUSCLE: multiple sequence alignment with high accuracy and high

throughput. *Nucleic Acids Res.* 32**,** 1792-1797. doi:10.1093/nar/gkh340

Gouy, M., Guindon, S., and Gascuel, O. (2010). SeaView version 4: a multiplatform graphical user interface for sequence alignment and phylogenetic tree building. *Mol. Biol. Evol.* 27**,** 221-224. doi:10.1093/molbev/msp259

Guindon, S., Dufayard, J-F., Lefort, V., Anisimova, M., Hordijk, W., and Gascuel, O. (2010). New algorithms and methods to estimate maximum-likelihood phylogenies: assessing the performance of PhyML 3.0. *Syst. Biol.* 59**,** 307-321. doi:10.1093/sysbio/syq010

Le, S.Q., and Gascuel, O. (2008). An improved general amino acid replacement matrix. *Mol. Biol. Evol* 25**,** 1307-1320. doi:10.1093/molbev/msn067

Pereira-Leal, J.B., Abreu, I.A., Alabaça, C.S., Almeida, M.H., Almeida, P., Almeida, T., Amorim, M.I., Araújo, S., Azevedo, H., Badia, A., et al. (2014). A comprehensive assessment of the transcriptome of cork oak (*Quercus suber*) through EST sequencing. *BMC Genomics* 15**,** 371. doi:10.1186/1471-2164-15-371

Pfaffl, M.W. (2001). A new mathematical model for relative quantification in real-time RT–

PCR. *Nucleic Acids Res.* 29**,** e45. doi:10.1093/nar/29.9.e45

Van Bel, M., Proost, S., Wischnitzki, E., Movahedi, S., Scheerlinck, C., Van de Peer, Y.,

and Vandepoele, K. (2012). Dissecting plant genomes with the PLAZA comparative genomics platform. *Plant Physiol.* 158**,** 590-600. doi:10.​1104/​pp.​111.​189514

Ye, J., Coulouris, G., Zaretskaya, I., Cutcutache, I., Rozen, S., and Madden, T.L. (2012). Primer-BLAST: a tool to design target-specific primers for polymerase chain reaction.

*BMC Bioinformatics* 18**,** 13:134. doi: 10.1186/1471-2105-13-134.
